# ZBTB32 performs crosstalk with the glucocorticoid receptor and is crucial in glucocorticoid responses to starvation

**DOI:** 10.1016/j.isci.2021.102790

**Published:** 2021-06-28

**Authors:** Lise Van Wyngene, Tineke Vanderhaeghen, Ioanna Petta, Steven Timmermans, Katrien Corbeels, Bart Van der Schueren, Jolien Vandewalle, Kelly Van Looveren, Charlotte Wallaeys, Melanie Eggermont, Sylviane Dewaele, Leen Catrysse, Geert van Loo, Rudi Beyaert, Roman Vangoitsenhoven, Toshinori Nakayama, Jan Tavernier, Karolien De Bosscher, Claude Libert

**Affiliations:** 1 Center for Inflammation Research, VIB Center for Inflammation Research, 9000 Ghent, Belgium; 2 Department of Biomedical Molecular Biology, Ghent University, 9000 Ghent, Belgium; 3 Ghent Gut Inflammation Group (GGIG), Ghent University, 9000 Ghent, Belgium; 4 Department of Rheumatology, Ghent University, 9000 Ghent, Belgium; 5 Department of Chronic Diseases and Metabolism – Endocrinology, KU Leuven, Leuven, Belgium; 6 Department of Immunology, Graduate School of Medicine, Chiba University, Chiba, Japan; 7 Center for Medical Biotechnology, VIB Center for Medical Biotechnology, 9000 Ghent, Belgium; 8 Cytokine Receptor Laboratory (CRL), Department of Biomolecular Medicine, Faculty of Medicine and Health Sciences, Ghent University, 3 Albert Baertsoenkaai, 9000 Ghent, Belgium; 9 Translational Nuclear Receptor Research Lab, Department of Biomolecular Medicine, Faculty of Medicine and Health Sciences, Ghent University, 3 Albert Baertsoenkaai,9000 Ghent, Belgium

**Keywords:** biological sciences, physiology, animal physiology, Endocrinology

## Abstract

The hypothalamic-pituitary-adrenal (HPA) axis forms a complex neuroendocrine system that regulates the body’s response to stress such as starvation. In contrast with the glucocorticoid receptor (GR), Zinc finger and BTB domain containing 32 (ZBTB32) is a transcription factor with poorly described functional relevance in physiology. This study shows that ZBTB32 is essential for the production of glucocorticoids (GCs) in response to starvation, since ZBTB32^−/−^ mice fail to increase their GC production in the absence of nutrients. In terms of mechanism, GR-mediated upregulation of adrenal *Scarb1* gene expression was absent in ZBTB32^−/−^ mice, implicating defective cholesterol import as the cause of the poor GC synthesis. These lower GC levels are further associated with aberrations in the metabolic adaptation to starvation, which could explain the progressive weight gain of ZBTB32^−/−^ mice. In conclusion, ZBTB32 performs a crosstalk with the GR in the metabolic adaptation to starvation via regulation of adrenal GC production.

## Highlights

• ZBTB32 is involved in the glucocorticoid production in response to starvation
• GR-mediated upregulation of adrenal *Scarb1* regulates cholesterol import
• The weight gain of ZBTB32^−/−^ mice is associated with aberrant metabolic adaptations

## Graphical abstract

**Figure:**
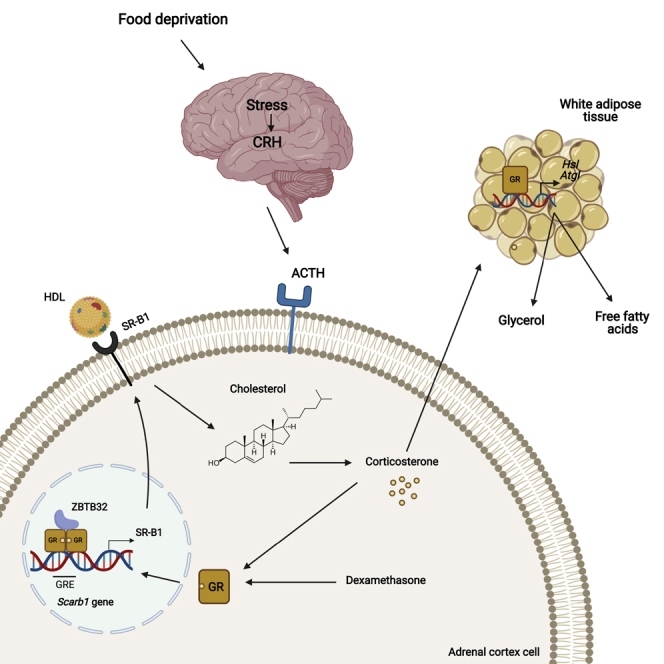

Nutrient shortage is sensed by the brain and causes the activation of the HPA axis. The synthesis and release of corticosterone by the adrenal glands is stimulated via the release of ACTH. Cholesterol, the precursor of corticosterone, is imported into the adrenal cortex cells via high-density lipoproteins (HDLs) which are internalized via the SR-B1 (Scarb1) receptor. ZBTB32 acts as a GR co-activator by enhancing the GR-induced upregulation of *Scarb1* gene expression and thereby controlling the cholesterol import into the adrenal glands during food deprivation. The expression of *Scarb1* is positively regulated by corticosterone and the GR agonist dexamethasone. Furthermore, corticosterone is also responsible for the metabolic adaptations of mice during starvation, by inducing lipolysis in the white adipose tissue (WAT) thereby releasing glycerol and free fatty acids into the bloodstream. Glycerol will then be used as a precursor for gluconeogenesis in the liver, while the FFAs will induce PPARα signaling in the liver. Lower corticosterone levels in ZBTB32^−/−^ mice are associated with disturbances in the metabolic adaptation to starvation, including lower lipolytic activity of WAT and aberrant PPARα signaling in the liver, which could result in weight gain and fat mass accumulation in ZBTB32^−/−^ mice over time.

## Introduction

Since the prevalence of obesity has tripled after 1975, obesity has been associated with an increased risk of disease and has been identified as an underlying cause of death in 20% of deaths in the USA (de Cosio et al., 2021). Obese individuals are not only at risk for chronic metabolic diseases, collectively referred to as the “metabolic syndrome”, including diabetes, fatty liver disease, and cardiovascular disease, but they are also vulnerable to acute diseases (such as sepsis) and cancer (Collaborators, 2018). Therefore, identification of molecules involved in regulation of body weight is of primary importance.

Under healthy conditions, the adipose tissue strictly fine-tunes the balance between triglyceride (TG) synthesis and lipid breakdown, creating a constant state of flux that is needed to meet physiological demands. Accordingly, obesity is the result of an imbalance between the rate of fat synthesis and fat catabolism in white adipose tissue (WAT) and lipid-metabolizing organs (Schweiger et al., 2017). This theory is supported by stable isotope studies demonstrating that TG synthesis is increased in the fat mass of obese individuals, while TG breakdown is decreased (Arner et al., 2011). In general, the excess energy stored as fat in adipose tissue can be exploited in times of food shortage, such as during fasting and starvation, or during intensive exercise. In addition, recent evidence suggests that a similar shift in oxidative metabolism occurs in the brain during sleep, when oxidation of fatty acids and ketone bodies (KBs) partly replace glucose as a cerebral energy source (Aalling et al., 2018).

Although being overweight is strongly linked with feeding habits and food quality, many genetic susceptibility loci have been identified in humans and mice. In the latter, two loci on chromosome 7 have been recurrently linked with body fat mass (Diament and Warden, 2004; Yi et al., 2004) i.e., a locus on distal chromosome 7 likely affecting expression of the uncoupling protein coding gene *Ucp2* (Surwit et al., 1998), and a locus on proximal chromosome 7 of unknown gene identity. Disturbances of endocrine systems have been identified as additional major players in the development of obesity and associated metabolic abnormalities (Pasquali et al., 2002). The hypothalamic-pituitary-adrenal (HPA) axis forms a complex neuroendocrine system that interacts directly via feedforward and feedback reactions to regulate the body’s response to stress. The activation of the HPA axis results in the conversion of cholesterol to glucocorticoids (GCs, corticosterone in rodents, cortisol in humans) and these GCs activate the GR which has important anti-inflammatory and metabolic functions (Habib et al., 2001; Sapolsky et al., 2000). The role of GCs in obesity is controversial: Chronic high levels of GCs can lead to obesity (John et al., 2016), (Smith, 1996) but, on the other hand, GCs stimulate lipolysis in the WAT (Van Wyngene et al., 2018).

The zinc finger and BTB domain containing 32 (ZBTB32) protein, also known as testis zinc finger protein (TZFP) or repressor of GATA (ROG), is a transcription factor belonging to the BTB/POZ-ZF protein family. It is encoded by the *Zbtb32* gene, located on proximal chromosome 7 in the mouse genome. ZBTB32 was shown to ensure proper regulation of meiosis during spermatogenesis by acting as a repressor of the androgen receptor (Furu and Klungland, 2013). Moreover, ZBTB32 is also involved in the regulation of the proliferative stages of primitive hematopoietic progenitors and lymphocytes, and absence of ZBTB32 results in increased T lymphocyte proliferation, cytokine production, and altered hematopoietic stem cell homeostasis in mice (Bilic and Ellmeier, 2007; Furu and Klungland, 2013; Piazza et al., 2004). So far, however, a link between ZBTB32 and regulation of endocrine systems has not been demonstrated.

In this study, we demonstrated that the absence of ZBTB32 in whole-body knockout mice (ZBTB32^−/−^) leads to progressive weight gain and dysfunctional production of GCs by the adrenal glands in response to starvation. We identified ZBTB32 as an interaction partner of the GR and demonstrated an essential role for ZBTB32 as a GR co-activator during stress-induced cholesterol import into the adrenal cells via upregulation of *Scarb1*. In conclusion, we have identified a metabolic regulator of GC signaling that when compromised could contribute to progressive weight gain through alterations of the endocrine system and physiological metabolic adaptation.

## Results

### ZBTB32^−/−^ mice are heavier and have more fat mass

**Figure 1:**
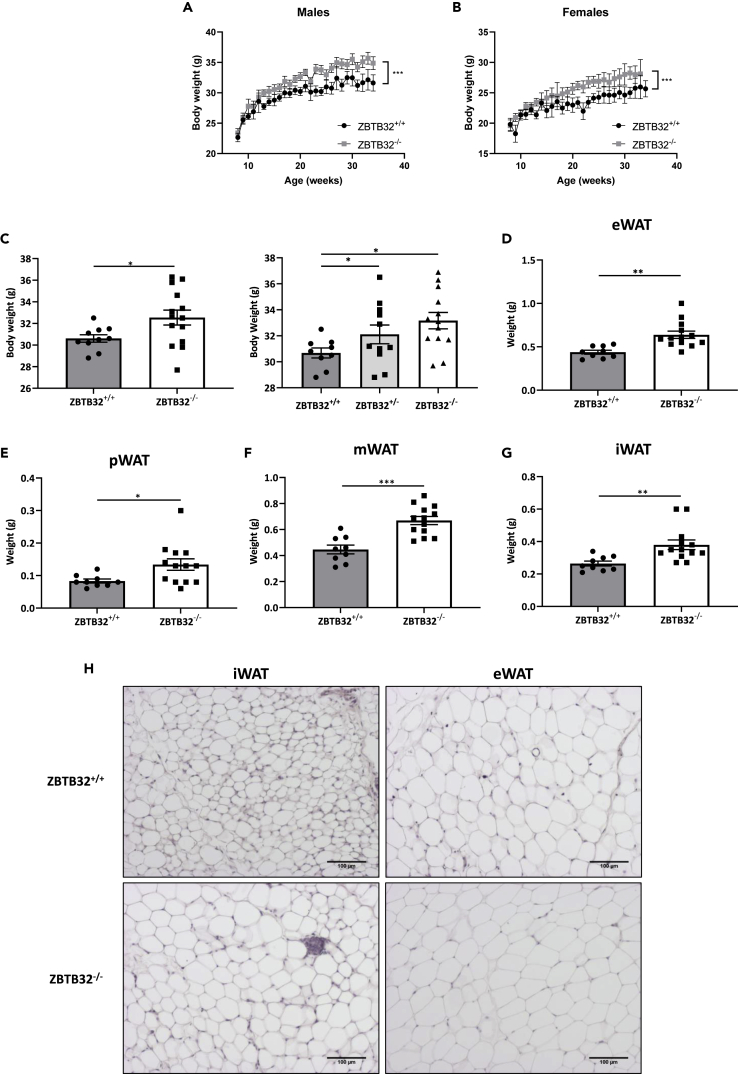
ZBTB32^−/−^ mice have higher body weight and fat mass (A**-**B) Body weights of (A) male and (B) female ZBTB32^+/+^ and ZBTB32^−/−^ mice between the ages of 8 weeks and 34 weeks. p values were <0.001 and calculated as described in Quantification and Statistical Analysis in the STAR Methods section. (C–G) Male ZBTB32^+/+^ and ZBTB32^−/−^ mice were aged to 25 weeks, after which (C) body weight was measured and weight of (D) epididymal white adipose tissue (eWAT), (E) perirenal WAT (pWAT), (F) mesenteric WAT (mWAT), and (G) inguinal WAT (iWAT) was determined. (C) In an additional experiment, the body weight of ZBTB32^+/−^ males was also measured. p values were calculated using one-way ANOVA. (H) Histological analysis via hematoxylin and eosin (H&E) staining of iWAT and eWAT paraffin sections of ZBTB32^+/+^ and ZBTB32^−/−^ mice at the age of 25 weeks. Values are shown as mean ± SEM, p values were calculated via two-way Student’s t-tests except if otherwise stated (n = 10-15 mice/group, pooled data of 2 independent experiments). ∗∗∗∗p< 0.0001; ∗∗∗p< 0.001; ∗∗p < 0.01; ∗p< 0.05.

**Figure 2:**
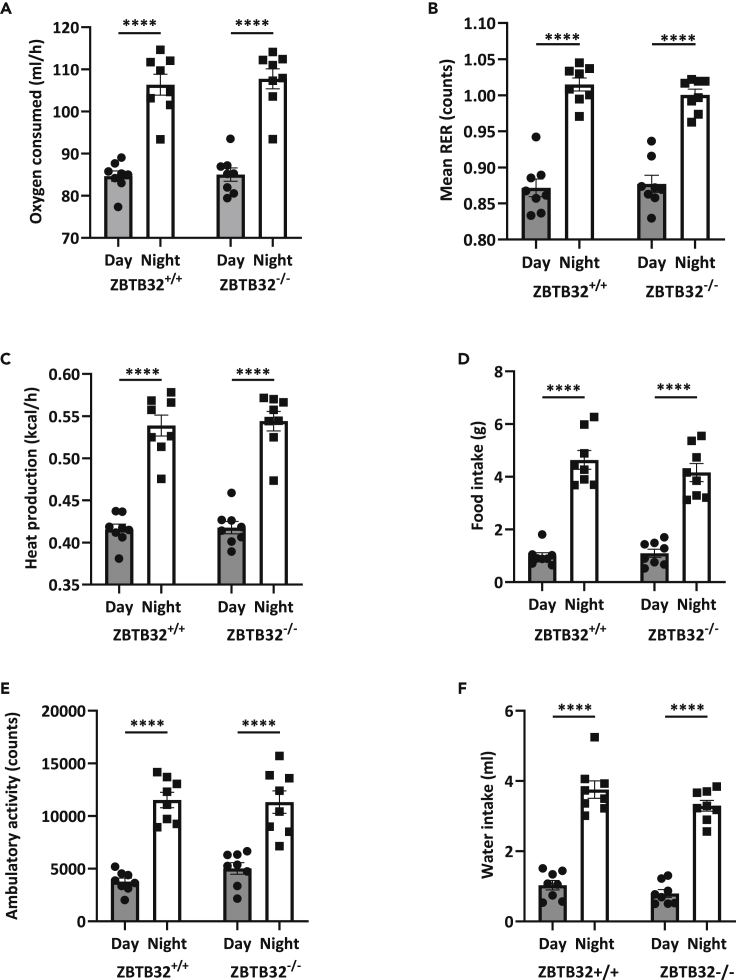
No change in energy expenditure between ZBTB32^+/+^ and ZBTB32^−/−^ mice Mice were studied in metabolic cages and the following basic parameters were measured during the daytime and at night: (A) oxygen consumption, (B) mean respiratory exchange ratio (RER), (C) heat production, (D) food intake, (E) ambulatory activity and (F) water intake. All parameters increase significantly during the night, but no differences between ZBTB32^+/+^ and ZBTB32^−/−^ mice were observed. Values are presented as mean ± SEM and p values were calculated via two-way ANOVA (n = 8 mice/group). ∗∗∗∗P < 0.0001.

ZBTB32^+/+^ and ZBTB32^−/−^ male and female mice were assessed for increase in body weight over a period of approximately 30 weeks, i.e., from ages 8 weeks until age 34 weeks (Figures 1A and 1B). Both males and females developed a significantly higher (both p < 0.001 see STAR Methods) body weight to an equal extent in the absence of ZBTB32. In more detail, at the age of 25 weeks, mice were assessed for body weight, body length, and weight of fat pads and liver. Again, ZBTB32^−/−^ mice displayed a significant increase in body weight and ZBTB32^+/−^ mice displayed an intermediate phenotype, suggesting a dose-response relationship (Figure 1C). This increase in weight is not attributed to an overall bigger stature of ZBTB32^−/−^ mice as both genotypes had similar body lengths, or to increased food intake (Figures S1A and S1B). In addition, liver weights were not different between ZBTB32^+/+^ and ZBTB32^−/−^ mice (Figure S1C). We assessed the weight of several visceral and subcutaneous fat pads (WAT) in age-matched ZBTB32^+/+^ and ZBTB32^−/−^ mice and demonstrated that epidydimal (eWAT), perirenal (pWAT), mesenteric (mWAT), and inguinal (iWAT) fat pad weights were significantly higher in 25-week-old ZBTB32^−/−^ mice compared to ZBTB32^+/+^ mice (Figures 1D–1G). Histological analysis with H&E staining demonstrated enlarged adipocytes in both eWAT and iWAT of ZBTB32^−/−^ compared to ZBTB32^+/+^ mice (Figure 1H). These data indicate that the increased body weight of ZBTB32^−/−^ mice is caused by an increment in adipocyte size and fat mass. To study the basic metabolic activity of ZBTB32^+/+^ and ZBTB32^−/−^ mice, metabolic cages were used to measure food and water uptake, oxygen consumption, respiratory exchange ratio, heat production and ambulatory activity (Figure 2). It was clear that both groups of mice increased all parameters at night, but no differences were detected between ZBTB32^+/+^ and ZBTB32^−/−^ mice, which was important to rule out energy expenditure as a basis of the body weight phenotype.

### ZBTB32^−/−^ adrenal glands produce less GCs upon starvation

**Figure 3:**
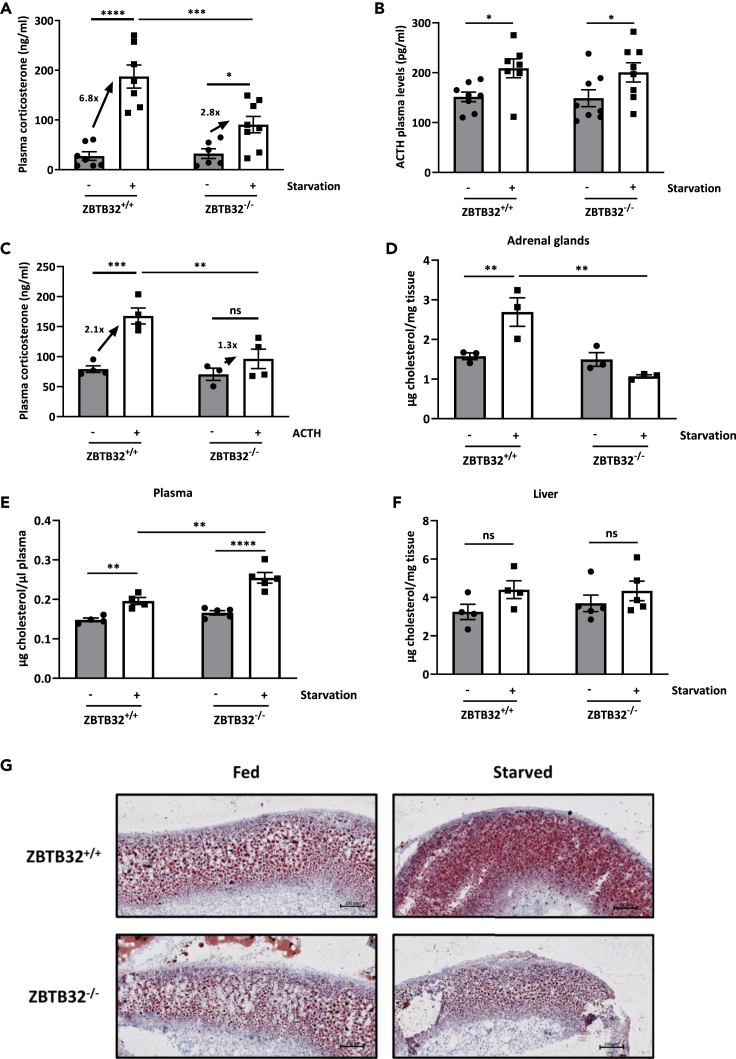
Absence of ZBTB32 leads to reduced GC production during starvation (A and B) ZBTB32^+/+^ and ZBTB32^−/−^ mice were fed *ad libitum* or starved for 24h. Blood was collected and plasma was obtained to measure (A) GC levels and (B) ACTH levels. (C) Mice were injected with native ACTH protein, and blood and plasma were collected 60 min after injection to measure GC levels. (D–F) Mice were fed *ad libitum* or starved for 24h after which blood and adrenal glands were isolated. Cholesterol levels in (D) adrenal homogenates, (E) plasma, and (F) liver homogenates were quantified. Values are presented as mean ± SEM and p values were calculated via two-way ANOVA tests (n = 6/8 mice/group, data representative of 2 independent experiments). ∗∗∗∗p< 0.0001; ∗∗∗p< 0.001; ∗∗p< 0.01; ∗p< 0.05. (G) Representative pictures of adrenal cortexes of ZBTB32^+/+^ and ZBTB32^−/−^ mice fed *ad libitum* or starved for 24h (n = 5) and stained with Oil Red O.

To investigate the molecular drivers behind the steady weight gain in ZBTB32^−/−^ mice, the functionality of the HPA axis during food deprivation was determined after 24h fasting in ZBTB32^+/+^ and ZBTB32^−/−^ mice. The major motivation to study the GC levels in this study is that besides glucagon and epinephrine, they are the major activators of lipolysis in WAT (Oprea et al., 2019) and, moreover, we have collected convincing evidence that ZBTB32 performs protein-protein interaction with GR (see further). Since the adrenal glands are the main producers of GCs, we studied the GC levels after starvation in the plasma of ZBTB32^+/+^ and ZBTB32^−/−^mice. Upon starvation, ZBTB32^+/+^ mice displayed a significant induction of plasma GC levels which was, however, less prominent in ZBTB32^−/−^ mice, despite similar adrenocorticotropic hormone (ACTH) levels in both genotypes, suggesting that ZBTB32 is an important mediator in the starvation-induced GC release (Figures 3A and 3B). Furthermore, intraperitoneal injection of 5 μg/g ACTH was unable to induce an increase in circulating GC levels in ZBTB32^−/−^ mice 60 min post-ACTH injection (Figure 3C).

GC synthesis by the adrenal gland requires uptake of the precursor cholesterol through internalization of lipoprotein particles from the blood (Kraemer, 2007). After a 24h starvation, adrenal glands of ZBTB32^+/+^ mice contained significantly increased amounts of free cholesterol, while absence of ZBTB32 prevented the increase of cholesterol in the adrenal glands (Figure 3D). In blood, circulating levels of cholesterol were increased in both ZBTB32^+/+^ and ZBTB32^−/−^ mice after starvation, however, levels were significantly higher in the blood of ZBTB32^−/−^ mice compared to ZBTB32^+/+^ mice (Figure 3E). To exclude aberrations in cholesterol synthesis by the liver, cholesterol levels and expression of key genes involved in hepatic cholesterol synthesis (starting from acetyl-CoA), were determined 24h after removal of food. Slightly increased levels of free cholesterol could be observed in the livers of both ZBTB32^+/+^ and ZBTB32^−/−^ mice after starvation, although these differences were not significant between treatments or genotypes (Figure 3F). Since the cholesterol measured in the adrenal gland is largely stored in the form of cholesterol esters, we measured the total cholesterol esters in adrenals, as well as in liver and plasma. These levels confirm that it is cholesterol uptake from the plasma that is reduced in ZBTB32^−/−^ mice during starvation (Figure S2). mRNA expression levels of *Acat2*, *Mvk,* and other essential genes of the cholesterol synthesis pathway in liver and adrenals of ZBTB32^−/−^ and ZBTB32^+/+^ mice were measured by RT-qPCR. In both organs, the mRNA expression levels of these genes were similar in both ZBTB32^+/+^ and ZBTB32^−/−^ mice in fed conditions. During starvation, the expression level of these genes declined in both ZBTB32^+/+^ and ZBTB32^−/−^ mice to the same extent (Figures S3 and S4). Since there were slightly increased levels of free cholesterol in the liver in both ZBTB32^+/+^ and ZBTB32^−/−^ mice (Figure 3F), this suggests that the absence of ZBTB32 does not lead to a defect in cholesterol synthesis, but rather to a problematic uptake of cholesterol into the adrenal gland during starvation. Finally, Oil Red O was used to stain lipids and cholesterol in the cortex of the adrenal glands of fed and 24h starved ZBTB32^+/+^ and ZBTB32^−/−^ mice. As expected, an increased signal, compared to the fed status, was observed in starved ZBTB32^+/+^ but not in ZBTB32^−/−^ mice (Figure 3G).

### ZBTB32 acts as a co-activator of the GR to facilitate cholesterol import into the adrenal gland during starvation

**Figure 4:**
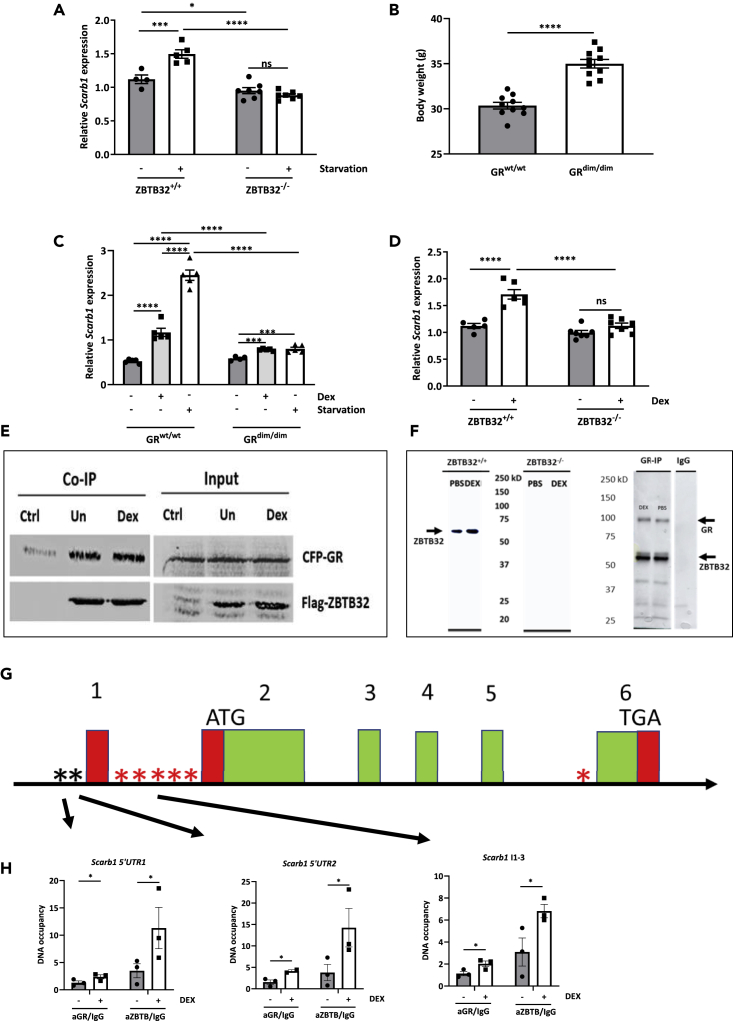
ZBTB32 functions as a co-activator of the GR to induce *Scarb1* expression (A) ZBTB32^+/+^ and ZBTB32^−/−^ mice were fed *ad libitum* or starved for 24h after which adrenal glands were isolated and expression of *Scarb1* was analyzed via RT-qPCR (n = 4-7 mice/group, data representative of 2 independent experiments). (B) Body weight of GR^wt/wt^ and GR^dim/dim^ mice at the age of 25 weeks (n = 10 mice/group, pooled data of 2 independent experiments). (C) GR^wt/wt^ and GR^dim/dim^ mice were starved for 24h or injected with Dex and 2h later adrenal glands were isolated and *Scarb1* expression was determined via RT-qPCR. (n = 4-5 mice/group, data representative of 2 independent experiments). Values normalized to the expression of the housekeeping genes *Hprt* and *Rpl*. (D) ZBTB32^+/+^ and ZBTB32^−/−^ mice were injected with Dex after 2h adrenal glands were isolated and expression of *Scarb1* was analyzed via qPCR (n = 5-7 mice/group, data representative of 2 independent experiments). (E) The interaction of GR with ZBTB32 was discovered by MAPPIT technology and here confirmed with co-immunoprecipitation as described in the STAR Methods section. HEK293T cells were transfected with flag-ZBTB32 and CFP-GR and stimulated either with vehicle (untreated control denoted ‘un’) or with 1 μM Dex for 2h. Cells were lysed and immunoprecipitated (IP) with M2-flag beads. Over-expression of CFP-GR with M2-flag beads incubation was used as negative control for the IP assay. Precipitates were immunoblotted with anti-GR and anti-Flag antibodies. (F) Left panel: Western blot for ZBTB32 in adrenals of ZBTB32^−/−^ and ZBTB32^+/+^ mice. For the detection of ZBTB32 on protein level, total protein was isolated from snap frozen adrenal glands (both adrenal glands were pooled per mouse) and 50 μg was loaded on a polyacrylamide gel and blotted. The blots were probed using a rabbit polyclonal antibody, specifically validated to recognize ZBTB32 in mouse adrenals. The antibody recognized ZBTB32 (53 kDa) in ZBTB32^+/+^ but not in ZBTB32^−/−^ mice. Two representative samples shown, one of a mouse injected with PBS, another one of a mouse injected (2h earlier) with 200 μg Dex. Full blot see Figure S11. Right panel: GR and ZBTB32 by Western blot after immunoprecipitation of GR using anti-GR antibodies. Mice were injected with PBS and with Dex (200 μg) and 2h later adrenals removed and processed as described in the STAR Methods section. Two representative samples are shown and a sample after IgG (instead of anti-GR IP) and full blots, including input samples and IgG controls are shown in Figure S11. (G) ChIP-SEQ-derived peaks of GR of PBS- or Dex-injected mice. Strong Dex-induced peaks were found at the intron 5 – exon 6 boundary (left) and in intron 1 (right). (H) Recruitment of GR to the *Scarb1* promotor ChIP-qPCR on adrenals glands of ZBTB32^+/+^ and ZBTB32^−/−^ mice 2h after injection of Dex or PBS (see STAR Methods section). Immunoprecipitated chromatin was analyzed by qPCR using the indicated primers (Table S1) for the *Scarb1* promotor. Values are shown as mean ± SEM. p values were determined via two-way Student's t-tests or two-way ANOVA tests. ∗∗∗∗p< 0.0001; ∗∗∗p< 0.001; ∗∗p< 0.01; ∗p< 0.05.

Cholesterol can be taken up by adrenal glands through the internalization of low-density lipoproteins (LDL) or high-density lipoproteins (HDL) via binding to their respective receptors on the adrenal cells. To investigate why the import of cholesterol into the adrenal glands would be dysfunctional in ZBTB32^−/−^ mice, we determined the mRNA expression of the LDL receptor (*Ldlr*) and the HDL receptor (*Scarb1*), in adrenal glands of ZBTB32^+/+^ and ZBTB32^−/−^ mice, in fed conditions and 24h post-starvation. Absence of ZBTB32 in adrenal glands had no major impact on *Ldlr* expression, nevertheless, *Ldlr* levels were lower in ZBTB32^−/−^ adrenal glands 24h after starvation compared to the levels in ZBTB32^+/+^ mice (Figure S5). In contrast, while ZBTB32^+/+^ adrenal glands show significantly upregulated *Scarb1* expression by starvation, likely leading to an increased uptake of cholesterol, such upregulated expression was not observed in ZBTB32^−/−^ adrenal glands. In addition, *Scarb1* expression was slightly (but significantly) lower in ZBTB32^−/−^ mice compared to ZBTB32^+/+^ mice that were fed (Figure 4A). Genes involved in GC synthesis (*Hsd11b1*, *Hsd11b2*, *Cyp11a1*, and *Cyp21a2*) were also measured by RT-qPCR to exclude aberrations during GC synthesis (Figure S6). The mRNA expression levels of these genes generally declined 24h after starvation, and the final levels (after starvation) were never lower in ZBTB32^−/−^ mice compared to ZBTB32^+/+^ mice.

While studying the mechanism of induction of *Scarb1* during starvation, we found that the GR, encoded by the *Nr3c1* gene is involved. Indeed, GR^dim/dim^ mice, which express a point-mutant version of GR that compromises GR in its homodimerizations and DNA binding and thereby influences the expression of numerous genes (Reichardt, 1998), were found to display a mild overweight phenotype compared to GR^wt/wt^ littermates (Figure 4B). Moreover, the induction of the expression of *Scarb1* in the adrenal glands of GR^dim/dim^ mice after treatment with Dexamethasone (Dex), a powerful GR agonist, and upon starvation is significantly reduced, suggesting that *Scarb1* could be a GR-responsive gene (Figure 4C). Indeed, expression of *Scarb1* was shown to be upregulated in ZBTB32^+/+^ adrenal glands after Dex injection, however, such upregulation could not be observed in adrenal glands of ZBTB32^−/−^ mice (Figure 4D), indicating that the defect in *Scarb1* gene activation in response to starvation is not the result of a defect at the step of GC induction itself, but of a failure of the GR to respond to GCs. This defect in GR-induced expression is specific for *Scarb1*, since expression of other GR-responsive genes, including hepatic *Ddit4* and *Sgk1* and adrenal *Dusp1*, did not differ in ZBTB32^−/−^ mice upon Dex stimulation (Figure S7). Together, these data identify adrenal *Scarb1* as a GR-responsive gene and propose a role for ZBTB32 as a potential GR co-activator that is crucial for GR-induced cholesterol import during starvation through the upregulation of *Scarb1* gene expression and its subsequent functional presence on the surface of adrenal cells.

### GR interacts with ZBTB32 and binds to the *Scarb1* promotor

As a transcription factor, GR is known to interact with co-factors that influence its actions (Petta et al., 2016). Using Mammalian Protein-Protein Interaction Trap (MAPPIT) (Guiñez and Castilla, 1999), a two-hybrid based technology to identify protein-protein interactions (schematically outlined in Figure S8A) (Lievens et al., 2012), ZBTB32 was shown to interact with the GR in the absence of ligand, an interaction which is further enhanced after stimulation with Dex. The MAPPIT technology is based on transient expression of a bait (GR) and an array of preys (human ORFeome collection V5.1, consisting of 12,000 human genes) (Petta et al., 2017). We identified many known GR interaction partners (for example HSP90), confirming the validity of the MAPPIT technology, but several new ones were additionally picked up. For a full list of all discovered GR-partners, including known-partners, see Table S3. These interactions were further validated in 3 independent MAPPIT experiments and were thus considered as *bona fide* GR interactions. One such experiment is outlined in Figure S8B. HEK293T cells were transfected with a GR bait cDNA and with ZBTB32 prey cDNA and cells not stimulated and Dex stimulated and the intensity of the ZBTB32-GR interaction measured by a Luciferase reporter (see STAR Methods). The increase in Luciferase indicates strongly increased interaction between GR and ZBTB32 in the solution thus depending on GR-ligand binding, and therefore conceivably based on the conformational changes that Dex induces on GR, exposing the helix 12 which is essential for binding of transcriptional co-regulators.

In a second approach, the protein-protein interaction between GR and ZBTB32 was further confirmed by co-immunoprecipitation (co-IP) experiments: HEK293T cells were transfected with 3 μg flag-ZBTB32 and 4 μg of CFP-GR and stimulated either with vehicle (un) or with 1 μM Dex for 2h. Cells were lysed and an IP was performed with M2-flag beads. Over-expression of CFP-GR with M2-flag beads incubation was used as negative control (Ctr) for the IP assay. Precipitates were immunoblotted with anti-GR and anti-Flag antibodies (Figure 4E). Third, an anti-ZBTB32 polyclonal antibody (see STAR Methods) was able to detect ZBTB32 on Western blot of adrenal lysates of ZBTB32^+/+^ mice and not ZBTB32^−/−^ mice (Figure 4F left panel &Figure S11A). There was no obvious difference in ZBTB32 levels 2h after injection of Dex or PBS treatment of mice. A GR IP using the H300 antibody (Figure 4F right panel &Figure S11B) led to GR signal as well as ZBTB32 on Western blot suggesting co-IP of both proteins. No signals were found when a control IgG was used instead of H300. Fourth, the impact of ZBTB32 on GR transcriptional activity was studied in A549 human lung epithelial cells, which express GR and have a stably integrated GRE-Luciferase element inserted in the genome. Transfection of ZBTB32 full length cDNA in these cells caused a significantly higher induction of Luciferase by Dex incubation in a dose-responsive way (Figure S8C). Since this reporter construct is strictly dependent on a GR-responsive element and contains no ZBTB32 responsive element, these data suggest that the effect of ZBTB32 on GR function is based on a direct protein-protein interaction.

Since Dex induces *Scarb1* expression, we studied GR-DNA binding on the *Scarb1* locus by two approaches. First, we performed a genome-wide ChIP-seq study (see STAR Methods) and found intense GR recruitment by Dex, on the boundary of intron 5 - exon 6 (Figure 4G) and five peaks in intron 1 (Figure 4G). Second, analysis of the promotor region of the *Scarb1* gene using the ConTraV3 application (Kreft et al., 2017) identified the presence of two additional potential GR-DNA binding sites in the mouse *Scarb1* promotor. ChIP-qPCR analysis confirmed GR-DNA binding in adrenal glands of ZBTB32^+/+^ mice upon Dex stimulation to this region in the genome that contains 2 adjacent GR-DNA binding sequences (Figures 4H and S8D). Importantly, GR-DNA binding was still observed in the absence of ZBTB32, suggesting that ZBTB32 is dispensable for GR-DNA binding to the *Scarb1* promoter. Together, these results demonstrate that the GR interacts with ZBTB32 and that GR binds to the *Scarb1* promotor to positively regulate *Scarb1* expression.

### Metabolic adaptations to starvation are impaired in the fat tissue of ZBTB32^−/−^ mice

**Figure 5:**
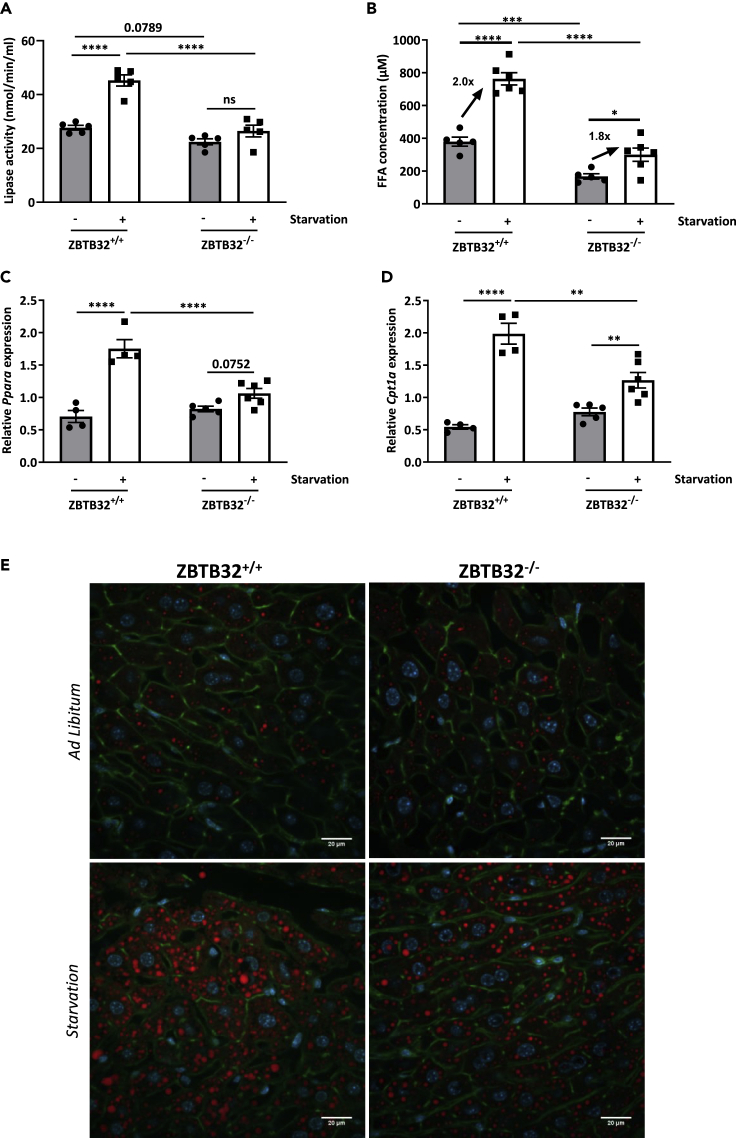
Defective GC production leads to metabolic dysfunction during starvation in ZBTB32^−/−^ mice ZBTB32^+/+^ and ZBTB32^−/−^ mice were fed *ad libitum* or starved for 24h (n = 5-7 mice/group, data representative of 2 independent experiments). (A) eWAT was isolated and lipolytic activity was determined. Values are represented as mean lipase activity/ml WAT homogenate ±SEM. (B) Free fatty acid levels in plasma. (C and D) Liver was isolated and mRNA expression levels of (C) *Ppara* and (D) *Cpt1a* were determined via RT-qPCR. Data are normalized to the expression of the housekeeping genes *Hprt* and *Rpl*. (E) Immunofluorescent images of livers visualizing lipid droplets (LDs). Cryosections were stained with Acti-stain (green), Hoechst (blue), and LipidTOX (red). Z-stacks were generated in 5–10 areas scattered across the entire tissue section. Values are shown as mean ± SEM. p values were determined via two-way ANOVA tests. Scale bar = 20 μm. ∗∗∗∗p< 0.0001; ∗∗∗p< 0.001; ∗∗p< 0.01; ∗p< 0.05.

To investigate the consequence of the reduced GC production by the adrenal glands of ZBTB32^−/−^ mice during starvation, in relation to the increase in body weight and fat volume, we examined several aspects of fat catabolism, including lipolytic activity in fat tissue, circulating free fatty acid (FFA) levels, hepatic PPARα-target gene expression, and hepatic steatosis. During a physiological starvation response, lipolysis in WAT is enhanced through several mechanisms, including activation of lipases by GCs (Xu et al., 2009). Total lipolytic activity was significantly induced in eWAT of ZBTB32^+/+^ mice 24h after starvation, while this increase was strikingly not observed in eWAT of ZBTB32^−/−^ mice (Figure 5A). In addition, expression analysis of hormone-sensitive lipase (*Hsl)* and adipose triglyceride lipase (*Atgl)*, the two major lipases, revealed a strong upregulation of *Hsl* expression in eWAT of ZBTB32^+/+,^ mice, but not in eWAT of ZBTB32^−/−^ mice (Figure S9A). In contrast, *Atgl* expression was significantly upregulated in ZBTB32^−/−^ eWAT, however, significantly less expressed in eWAT of ZBTB32^+/+^ mice (Figure S9B).

Following the activation of lipolysis in fat tissue during starvation, FFA levels are increased in the bloodstream and are subsequently taken up by peripheral tissues for energy production (Cahill, 1970). Blood FFA levels were two-fold and significantly increased in ZBTB32^+/+^ mice after 24h of starvation (Figure 5B), while blood FFA levels were increased in ZBTB32^−/−^ mice (1.8 fold), however, were lower in fed conditions, and remained significantly lower upon starvation compared to the levels in ZBTB32^+/+^ mice (Figure 5B).

PPARα is the major transcription factor involved in adaptation to food deprivation and acts as a nutritional sensor by activation of fat metabolic pathways, such as β-oxidation and ketogenesis, predominantly in liver (Hashimoto et al., 2000). Contribution of GR in the function of PPARα has been found (Bougarne et al., 2018, 2019; Ratman et al., 2016). As FFAs are endogenous ligands for PPARα activation, we next investigated the transcriptional activity of PPARα in livers of ZBTB32^+/+^ and ZBTB32^−/−^ mice after 24h starvation. Expression levels of *Ppara*, *Cpt1a* and other PPARα-responsive genes involved in the β-oxidation pathway were strongly induced by starvation in the livers of ZBTB32^+/+^ mice, but less in the liver of ZBTB32^−/−^ mice upon starvation (Figures 5C, 5D, S9C, and S9D), suggesting that the metabolic adaptation to starvation via PPARα is impaired in ZBTB32^−/−^ mice due to the lack of endogenous FFA substrates. A direct interaction or co-factor role of ZBTB32 to PPARα cannot be excluded and needs further investigation in future studies.

Non-pathogenic hepatic steatosis is a physiological response to starvation, and lipids, released by the adipose tissue through lipolysis, are taken up by hepatocytes and safely stored in lipid droplets (LDs) (Herms et al., 2013). Hepatic steatosis in livers of ZBTB32^+/+^ and ZBTB32^−/−^ mice 24h after starvation was assessed through LipidTOX staining and through quantification of the amounts and the size of individual LDs. A 24h starvation significantly increased the amount and size of LDs/cell in livers of ZBTB32^+/+^ mice (Figures 5E,S9E, and S9F). An increase in hepatosteatosis was also observed in livers of ZBTB32^−/−^ mice 24h after starvation, however, the amounts of LDs was lower compared to ZBTB32^+/+^ mice, while the average size of LDs did not differ between both genotypes (Figures 5E,S9E, and S9F).

### Impact of ZBTB32 on glycemic control in mice

**Figure 6:**
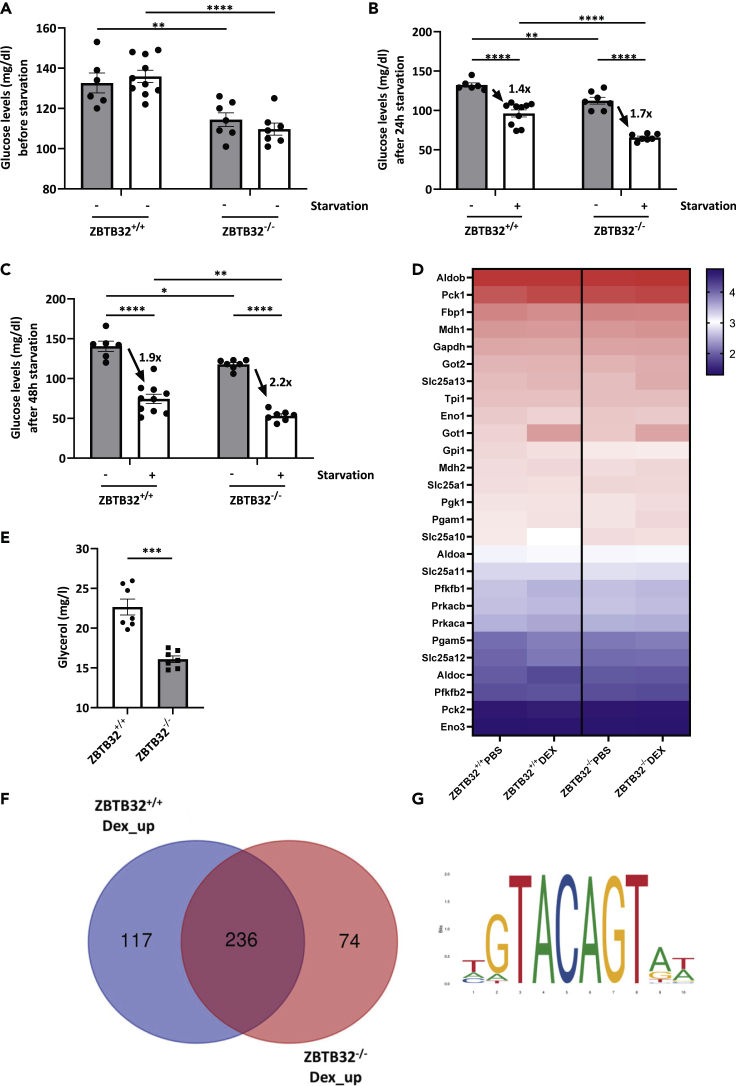
ZBTB32^−/−^ mice have low blood glucose in fed and in starved conditions (A–C) Blood glucose levels were measured via the tail vein of ZBTB32^+/+^ and ZBTB32^−/−^ mice prior to starvation (A), and 24h (B) and 48h (C) after starvation (n = 6-10/group). (D) Heatmap showing the log_10_ expression levels of 27 gluconeogenesis-controlling genes, in livers of ZBTB32^+/+^ and ZBTB32^−/−^ mice treated with PBS or Dex and 2h later measured by RNA-seq (n = 3 per group). Genes are ranked from top to bottom according to expression level in ZBTB32^+/+^ PBS mice. (E) Glycerol levels in plasma of ZBTB32^+/+^ and ZBTB32^−/−^ male mice of 12 weeks old (n = 7 per group). p values were calculated using Student’s t-test. (F) Venn-diagram displaying the numbers of significant (FDR %) and sufficient (LFC 0.8) Dex-induced genes as found by the RNA-seq experiment. (G) The ZBTB32 responsive element that was investigated in the genes displayed in the Venn-diagram. Values are represented as mean ± SEM. p values were determined via two-way ANOVA, except if otherwise stated. ∗∗∗∗p< 0.0001; ∗∗∗p< 0.001; ∗∗p< 0.01; ∗p< 0.05.

Finally, starvation leads to mild hypoglycemia (Kuo et al., 2015), due to fast consumption of glycogen. Blood glycerol, produced from the WAT by lipolysis (stimulated by GCs), as well as certain amino acids produced by proteolysis in muscle, are imported in the liver and will undergo gluconeogenesis, which is known to be strongly coordinated by GR (Van Wyngene et al., 2018). Based on the reduced GC production during starvation in ZBTB32^−/−^ mice, we studied the control of glucose concentrations in blood during starvation. Therefore, blood glucose levels were measured via the tail vein in ZBTB32^+/+^ and ZBTB32^−/−^ mice prior to starvation and after starvation. Before starvation, blood glucose levels were already significantly lower in ZBTB32^−/−^ mice (Figure 6A). Moreover, blood glucose levels further declined in these mice as starvation proceeded (Figures 6B 24h after starvation onset and Figures 6C 48 h), showing that ZBTB32^−/−^ mice were less able to control the decline in blood glucose levels upon starvation (Figure 6). In view of the previously described data, we investigated the expression of genes involved in gluconeogenesis in liver. ZBTB32^+/+^ and ZBTB32^−/−^ mice were injected with PBS or with Dex and 2 h later, genome-wide transcriptomics was studied by RNA sequencing (RNA-seq). Using the normal filters, we found the expression of 27 of the 31 genes encoding proteins which are involved in this process (https://maayanlab.cloud/Harmonizome/gene_set/Gluconeogenesis/Reactome+Pathways). Despite some minor expression differences between ZBTB32^+/+^ and ZBTB32^−/−^ mice, both types of mice display equal amounts of these crucial genes in PBS or in Dex stimulated conditions, suggesting that GR functions fine in gluconeogenesis independent of ZBTB32 (Figure 6D). So, the problematic glucose control in ZBTB32^−/−^ mice during starvation is likely to be the result of a reduced serum level of the gluconeogenesis precursor glycerol, which was found to be the case (Figure 6E). In view of the data described in Figures 5A and 5B, this result confirms the poor lipolysis status in the ZBTB32^−/−^ mice.

Analyzing the RNA-seq data, after filtering on at least 10 transcripts/gene/cell, a list of 11,933 genes with reliable expression levels in livers of mice were considered further. After Dex stimulation, 353 genes and 310 genes were significantly upregulated (log fold change [LFC] > 0.8 and FDR <0.05) in the liver of ZBTB32^+/+^ and ZBTB32^−/−^ mice, respectively. Although 236 genes were induced by Dex in the liver of both genotypes, Dex significantly increased the expression of 117 genes in ZBTB32^+/+^ mice alone. In contrast, 74 genes were specifically induced by Dex in the absence of ZBTB32 (Figure 6F), which appear to be ZBTB32 repressive genes since GR can only induced these genes when ZBTB32 is absent. In the multitude of Dex-induced RNA-seq transcriptomics performed in liver in our laboratory (see for example (Dendoncker et al., 2019), these genes have never been observed as GRE genes, and they will need further investigation. The list of these genes (family of 117, 236 and 74 genes) is provided as Table S2, which allows to investigate enriched pathways and gene function by using the online tool Enrichr (https://maayanlab.cloud/Enrichr/). The table also gives a very minimal analysis (using Enrichr) of obvious pathways linked to the three lists of genes. A HOMER motif analysis (Heinz et al., 2010) was used for the identification of canonical ZBTB32 responsive elements (ZREs) and GR responsive elements (GREs) in the 1 kb region prior to the transcription start sites of these genes (Figure 6G). Such motifs were identified in 26/117 genes (22%), but also 12/74 genes (16%) induced by Dex in ZBTB32^−/−^ mice alone and 81/236 genes (34%) induced by Dex in both genotypes contained a ZRE. Thus, despite ZBTB32 absence or presence influencing the response of GR to Dex, we found no significant link to ZREs in the promotors of these genes. An influence of ZBTB32 on GR via protein-protein interaction might be a conceivable explanation, but the gene-specific aspect remains unexplained. HOMER identified canonical GREs in 14/117 (12%), 24/236 (10%) and 3/74 genes (4%). The latter GR-induced genes appear to be induced by a mechanism not involving direct GR-DNA binding. The enriched, known motifs identified via HOMER motif analysis are displayed in Figure S10: Obviously, the groups of 117 and 236 genes are characterized by classical GRE, but not the group of 74 genes.

Together, these data suggest that the dampened stress response in ZBTB32^−/−^ mice leads to an impaired metabolic adaptation to starvation, as demonstrated by the reduced lipolytic activity of ZBTB32^−/−^ fat pads and decreased PPARα transcriptional activity in livers of these mice. We hypothesize that these metabolic dysregulations could lead to the increased weight gain of ZBTB32^−/−^ mice over time through impairment of proper adaptation to the nutritional status (**Graphical abstract**).

## Discussion

The HPA axis forms a complex neuroendocrine system that interacts directly via feedforward and feedback reactions to regulate the body’s response to stress such as starvation. In particular, GCs produced by the adrenal cortex, have important effects on adipose tissue development and metabolism (Spencer and Tilbrook, 2011). Depending on the nutritional and hormonal environment, GCs can have stimulatory effects on adipocyte expansion or they can stimulate lipolytic activity. For example, during catabolic states such as fasting and starvation, GC synthesis is increased in the adrenal gland as a vital part of the stress response, and induces lipolysis to mobilize fat energy stores (Dallman et al., 1999). Chronic use of GCs leads to a redistribution of fat mass and a selective increase in abdominal fat (Pasquali et al., 1993; Smith, 1996). These observations have led to the concept that GCs play a role in the progression of obesity in man.

In rodents, which are nocturnal animals, peak GC levels are reached in the late afternoon-early night (Dickmeis, 2009). In adipose tissue, expression of adipose tissue triglyceride lipase gene (*Atgl*) and hormone-sensitive lipase gene (*Hsl*), are under control of several hormones (Shostak et al., 2013). We found that the deletion of the ZBTB32-coding gene (*Zbtb32*) leads to a progressive increased fat weight gain, slightly reduced circadian release of GCs, but also that ZBTB32 is required for an optimal synthesis of GCs during food deprivation-induced stress (starvation), leading to poor lipolysis in adipose tissue, and PPARα-induced signaling in the liver. We hypothesize that this suboptimal GC release to the changing nutritional status could be responsible for the weight gain and increased fat mass in adolescent ZBTB32^−/−^ mice. Indeed, a reduction of adrenal GC release, could progressively result in a lack of lipolysis, which leads to (1) less daily consumption of fat from the WAT, thus gradual relative increase in weight, (2) lower FFA and glycerol levels in the blood even in the normal fed condition and (3) lower FFA and glycerol levels in the starved condition, the latter leading to low gluconeogenesis in liver and thus lower blood glucose in the ZBTB32^−/−^ mice.

Scavenger receptor class B type 1 (SR-B1 or Scarb1) is an HDL receptor that is crucial for maintenance of free cholesterol in the adrenals, where it serves as the precursor for GC synthesis (Acton et al., 1996). GC levels of Scarb1-deficient mice were comparable to their wild-type counterparts under *ad libitum* feeding, and reduced plasma GC levels were observed during overnight fasting (Hoekstra et al., 2008). So, the role of Scarb1 in starvation-induced GC synthesis is crucial. In general, ZBTB32 deletion could be considered as a partial phenocopy of the Scarb1 deletion, as ZBTB32^−/−^ mice do not upregulate *Scarb1* expression, and do not increase their GC production to the same degree as ZBTB32^+/+^ mice, after starvation. Similar as Scarb1 deficiency, decreased levels of free cholesterol as well as cholesterol ester could be demonstrated in the adrenal glands of ZBTB32^−/−^ mice, most likely caused by impaired import of cholesterol. In contrast, we did not observe elevated blood ACTH levels in ZBTB32^−/−^ mice upon food deprivation, suggesting that deletion of ZBTB32 does not fully phenocopy the Scarb1-deletion, for which elevated blood ACTH was observed (Hoekstra et al., 2008).

Genes involved in the synthesis of GCs are often under positive and/or negative feedback control of GCs themselves, which act through the GR, as is the case for the corticotrophin-releasing hormone (*Crh)* (hypothalamus) and *Pomc* gene (pituitary), the latter of which encodes for the pro-opiomelanocortin prohormone, the precursor of ACTH (Drouin, 2016). Previous studies have demonstrated that adrenal *Scarb1* expression is subject to negative feedback regulation by GR. In *Crh*^−/−^ mice, a model of GC insufficiency, adrenal *Scarb1* expression was elevated basally, but decreased after oral administration of GCs (Mavridou et al., 2010). However, direct binding of GR to the *Scarb1* promotor could not be demonstrated by these authors. This is opposed by our data that demonstrate a positive effect of both Dex and starvation on *Scarb1* gene expression in the adrenal glands. Potentially, these differences could be attributed to the acute nature of the Dex stimulation in our study, compared to a chronic addition of GCs during 3 days in the *Crh*^−/−^ mouse model. In addition, one could argue that a constitutive knock out of the *Crh* gene and a starvation response might represent unique physiological states that result in differential transcriptional regulation of the *Scarb1* gene. Moreover, analysis of the *Scarb1* gene with a GR ChIP-seq identified six *bona fide* GR binding sites that are upregulated by Dex, and the ConTraV3 application (Kreft et al., 2017) identified the presence of two additional potential GRE sites, of which at least one could be bound in the adrenal gland by GR upon Dex stimulation, as we found by ChIP-qPCR. Finally, GR^dim/dim^ mice, which are poor in the induction of GR-homodimer-dependent genes, display poor Dex-induced as well as starvation-induced *Scarb1* gene expression. Although the GR^dim/dim^ mouse is not the ideal tool to study the GR-dimer dependent biological responses (Presman et al., 2014), it is currently the only one we have (Vandevyver et al., 2012, 2013). Our data confirm *Scarb1* as a *bona fide* GR responsive gene.

Based on our data, we propose a model in which ZBTB32 functions as a GR co-activator to induce the expression of *Scarb1* to facilitate the increase of GC synthesis via the enhanced uptake of cholesterol. The evidence of ZBTB32 as a GR-binding protein comes from several studies: (1) Based on the MAPPIT technique to search for GR-interaction proteins, starting with over 12.000 candidate cDNAs, we found ZBTB32, besides 31 others, including several expected GR partners, such as HSP90. (2) One-to-one MAPPIT experiments confirmed the interaction and (3) overexpression of tagged GR and ZBTB32 proteins confirmed this. (4) Finally, GR-IP from adrenal tissue was able to co-precipitate ZBTB32. From all protein-protein interaction technologies, MAPPIT is considered one of the most trustable for many reasons as outlined by Lavens et al. (2009) and Vyncke et al. (2019) (Lavens et al., 2009; Vyncke et al., 2019).

We found multiple GR-DNA binding sites at the *Scarb1* gene, several via experimental ChIP-seq, and two via prediction. Also, the GR-DNA binding to the two GREs of the *Scarb1* promotor was not affected by the absence of ZBTB32 and in the entire *Scarb1* locus, we found no convincing ZBTB32-binding sequence. It is plausible that ZBTB32 orchestrates transcription at the level of the co-activator complex at the *Scarb1* promotor rather than at the level of the GR-DNA interaction, as has been shown by several GR co-activators (Birth et al., 2017; Hill et al., 2016). Indeed, ZBTB32 overexpression was able to increase GR-transcriptional output at a GRE-Luc element, which lacked any ZBTB32 genomic binding element. Our analysis failed to detect convincing ZBTB32 binding sites in/around the *Scarb1* gene (Chou et al., 2010; Lin et al., 1999; Tang et al., 2001). A similar mechanism may apply to PPARα expression, which has been shown to be controlled by GCs and, given the importance of a direct crosstalk between GR and PPARα in the starvation response, a disabled PPARα pathway may as such additionally impact the obesogenic phenotype in absence of ZBTB32 (Bougarne et al., 2018, 2019; Ratman et al., 2016). By studying the Dex-induced (GR-mediated) transcriptional upregulation of genes in liver in ZBTB32^+/+^ and ZBTB32^−/−^ mice, we identified 74 genes induced by Dex regardless of ZBTB32. On the contrary, 117 genes require ZBTB32 for their gene induction, since these are not induced when ZBTB32 is absent. Based on HOMER motif analysis, the GRE was found to be enriched in this gene set, however no enrichment of the ZRE could be detected. These data may suggest that ZBTB32 collaborates with GR in a locus-specific way and via protein-protein interaction, not by DNA binding *per se*. This hypothesis should be tested by ZBTB32 ChIP experiments in the future.

In mice, the *Zbtb32* gene is located at the 30,6 MB position of proximal chromosome 7, a locus previously linked to obese phenotypes by QTL analysis in mice. The obese phenotype of the NZO/HlLtJ (New Zealand Obese) mice has been linked to a locus on proximal chromosome 7 (Taylor et al., 2001). In our recently released database of naturally occurring variant protein coding genes (mousepost.be (Timmermans et al., 2017)) across all 36 sequenced mouse inbred lines, we observe that the obese NZO/HlLtJ mice, compared to most other strains, express a mutant version of the *Zbtb32* gene, leading to a W226L mutation in the ZBTB32 protein, a mutation that, according to the Protein Variation Effect Analyzer (PROVEAN), has a high chance to impact protein function (Choi et al., 2012). These observations suggest that at least some obese phenotypes, maybe also in humans, are due to mutations in the gene coding for ZBTB32. Our data are derived from full-body ZBTB32^−/−^ mice, which is an inherent weakness in this study. Adrenal cortex-specific ZBTB32-deficient mice have not been generated so far. Nevertheless, in the future, such mice would be of great added value for this study. Equally important would be a way to supplement ZBTB32^−/−^ mice with extra corticosterone to complement for the reduced production in these mice and revert the heavier weight phenotype. Such experiments have been tried with corticosterone, but had to be stopped as this led to unacceptable stress as we published before (van Looveren et al., 2019).

In conclusion, we have identified ZBTB32 as an important player during starvation since the absence of ZBTB32 blunts the stress response to starvation due to impaired GC synthesis by the adrenal glands. We identified *Scarb1* as a *bona fide* GR-responsive gene and hypothesize that ZBTB32 acts as a GR co-activator in this context by enhancing the GR-induced upregulation of *Scarb1* expression and therefore in the control of cholesterol import into the adrenal glands during starvation. Consequently, failure to enhance the uptake of cholesterol via internalization of HDL most likely causes the lack of GC production by adrenal glands in ZBTB32^−/−^ mice. Lower GC levels in ZBTB32^−/−^ mice were associated with disturbances in the metabolic adaptation to starvation, including lower lipolytic activity of adipose tissue and aberrant PPARα signaling in the liver, which could result in weight gain and fat mass accumulation in ZBTB32^−/−^ mice over time.

### Limitations of the study

In this study, we have identified *Scarb1* as a GR responsive gene and that ZBTB32 can be described as a GR co-activator by enhancing the GR-induced upregulation of the *Scarb1* expression in response to starvation and thereby controlling the import of cholesterol into the adrenal glands. However, a weakness of our study is the use of full-body ZBTB32 knock-out mice. Therefore, it would be of great interest to generate adrenal cortex-specific ZBTB32-deficient mice to study the role of ZBTB32 specifically in the adrenal glands. Our data also highlight the importance of ZBTB32 in the GC response to starvation. Here, we have studied the GC response in both ZBTB32^+/+^ and ZBTB32^−/−^ mice upon nutrient deprived conditions. Ideally, the GC production needs to be investigated in ZBTB32^−/−^ mice and its wild-type littermates in basal conditions over time. Then, a clear role of ZBTB32 in the GC response could be linked with the weight gain observed in ZBTB32^−/−^ mice. It would also be interesting to study whether the heavier weight phenotype of ZBTB32^−/−^ mice could be compensated when these mice are supplemented with extra GCs. We believe that further studies are necessary to look further into the more detailed role of ZBTB32 in the adrenal glands specifically.

## STAR★Methods

### Key resources table

**Table:** 

REAGENT or RESOURCE	SOURCE	IDENTIFIER
**Antibodies**		

HCS LipidTOX™ Deep Red Neutral Lipid Stain for cellular imaging	Life Technologies Europe B.V.	CAT#H34477
Acti-stain 488 Phalloidin	Cytoskeleton Inc.	CAT#PHDG1
Rabbit polyclonal anti-Flag	Sigma	CAT#F7425
Rabbit polyclonal anti-GR	Santa Cruz Biotechnology	CAT#sc-8992; RRID:AB_2155784
Mouse monoclonal anti-GR	Santa Cruz Biotechnology	CAT#sc-393232
Goat polyclonal anti-rabbit IgG	Jackson ImmunoResearch laboratories	CAT#111-035-144; RRID:AB_2307391
Rabbit polyclonal anti-ZBTB32	Aviva Systems Biology	CAT#OACA06573
Rabbit HRP conjugated anti-IgG	R&D systems	CAT#HAF008
Normal rabbit IgG	Peprotech	CAT#500-P00

**Chemicals, peptides and recombinant proteins**		

ACTH native protein	Gentaur bvba	CAT#544-MBS142672
Rapidexon	Medini N.V.	/

**Critical commercial assays**		

Aurum total RNA mini kit	Bio-rad	CAT#732-6820
Sensifast cDNA synthesis kit	GC Biotech BV	CAT#BIO-650504
SensiFAST Sybr no-ROX mice	GC Biotech BV	CAT#CSA-01190
Cholesterol/cholesteryl Ester Assay Kit – Quantitation	Abcam	CAT#ab65359
Free Fatty Acid Assay Kit	Abnova	CAT#KA1667
Glycerol Colorimetric Assay Kit	Cayman Chemical	CAT#10010755
Corticosterone ELISA kit	Arbor Assays	CAT#K014-H1
ACTH ELISA kit	Antibodies-online	CAT#ABIN1113286
Lipase activity assay kit	Cayman Chemical	CAT#700640

**Deposited data**		

Raw and analyzed data RNA-seq	This paper	GSE176277
Raw and analyzed data ChIP-seq	This paper	GSE123953

**Experimental models: cell lines**		

Mouse hepatoma BWTG3 cells	This paper	In house: CB085
Human embryonic kidney T (HEK293T) cells	This paper	In house: CB10636

**Experimental models: organisms**		

Mouse: Zbtb32^tm1Iho^	Chiba University Japan	N/A

**Oligonucleotides**		

Primers for RT-qPCR, see Table S1	This paper	N/A
Primers for ChIP-qPCR, seeTable S1	This paper	N/A

**Recombinant DNA**		

Plasmid: pCLG-human GRa	This paper	N/A
Plasmid: pCLG-human ZBTB32	This paper	N/A
Plasmid: STAT3-responsive rPAP-Luciferase reporter	This paper	N/A
Plasmid: GRE-Luciferase reporter transfected with flag-ZBTB32	This paper	N/A
Plasmid: pMet7-CFP-GRa	This paper	N/A
Plasmid: pMet-Flag-ZBTB32	This paper	N/A

**Software and algorithms**		

Bowtie (v 1.0.0)	/	https://sourceforge.net/projects/bowtie-bio/files/bowtie/1.0.0/
HISAT v2.0.4	(Kim et al., 2015)	http://daehwankimlab.github.io/hisat2/
FeatureCounts	(Liao et al., 2014)	https://www.rdocumentation.org/packages/Rsubread/versions/1.22.2/topics/featureCounts
DESeq2	(Love et al., 2014)	https://bioconductor.org/packages/release/bioc/html/DESeq2.html
HOMER v4.6	(Heinz et al., 2010)	http://homer.ucsd.edu/homer/
GraphPad Prism software	/	https://www.graphpad.com/scientific-software/prism/

### Resource availability

#### Lead contact

Further information and requests for resources and reagents should be directed to and will be fulfilled by the lead contact, Claude Libert (claude.libert@irc.vib-ugent.be).

#### Material availability

This study did not generate new unique reagents.

#### Data and code availability

RNA-seq data: gene expression. Deposited at the National Center for Biotechnology Information Gene Expression Omnibus public database (http://www.ncbi.nlm.nih.gov/geo/) under accession number GSE176277. ChIP-seq data: accession number GSE123953.

### Experimental model and subject details

#### BWTG3 cell line

BWTG3 cells (mouse hepatoma cells) were maintained and grown in Dulbecco’s modified Eagle’s medium (DMEM) (house-made) containing 10% FCS, 1 mM sodium pyruvate, 0.1 mM nonessential amino acids, and 2 mM L-glutamine. Cells were starved for 24 h in Opti-MEM medium (Gibco, Invitrogen) prior to the start of the experiment. Cells were then exposed to 1 μM Dex (Sigma, D-2915).

#### Mouse models

ZBTB32^−/−^ mice, in a C57BL/6 background, were kindly provided by the laboratory of Professor Toshinory Nakayama (Chiba University, Japan). ZBTB32^−/−^ mice were backcrossed to wild type C57BL/6J mice, purchased from Janvier (Le Genest-St.Isle, France), to establish ZBTB32^−/−^ and ZBTB32^+/+^ mice by ZBTB32^+/−^ intercrosses. All mice were housed in light controlled (14 h light; 10 h dark), air-conditioned, conventional conditions with food and water *ad libitum*. All experiments were approved by the institutional ethics committee for animal welfare of the Faculty of Sciences, Ghent University, Belgium. The methods were carried out in accordance with the relevant guidelines and regulations. All male and female mice were used at 8–12 weeks, except for weight follow-up experiments. For these experiments, mice were aged to 25 or 35 weeks, after which organs were isolated, weighed and body length (from nose to the tail base) was determined. During starvation experiments, food was taken away for a period of 24h, starting from 8 am to 8 am the next day. GR^dim/dim^ mice were generated by H.M. Reichardt et al. and kept on an FVB/N background (Reichardt et al., 1998). Heterozygous GR^dim/WT^ mice were intercrossed to generate GR^WT/WT^ and GR^dim/dim^ homozygous mutant mice and housed in conditions identical to the ZBTB32 mice.

#### Injections and sampling

All injections were given intraperitoneally, and injections volumes were always adapted to the body weight of the mice. Blood was taken via retro-orbital bleeding after sedation via inhalation of isoflurane and collected in EDTA-coated tubes (Sarstedt Bvba) for plasma isolation. Plasma was prepared by centrifugation at 3,000 rpm for 15 min and stored at −20°C. For sampling of organs, mice were anesthetized by cervical dislocation at indicated timepoints. Samples for RNA extraction were stored in RNA Later (Life Technologies) at −20°C. Organs for cholesterol analysis were snapfrozen and stored at −20°C. For histology, fat pads were fixed in 4% paraformaldehyde and embedded in paraffin by a standard protocol and stained with hematoxylin and eosin (H&E). For the measurement of GC levels, mice were quickly bled via retro-orbital eye bleeding. This was done mouse by mouse in the animal house lab, to prevent stress induction with other mice.

### Method details

#### Energy expenditure study

Mice (8–14 weeks old) were housed individually in automated calocages for indirect calorimetry (PhenomasterCalocages TSE systems, Germany) on a 12h/12h light/dark cycle at 21°C with *ad libitum* access to food and water. Prior to actual measurements, mice were acclimatized to single housing and specific drinking bottles for 1 week. The body weight of the mice was assessed prior to the start of the measurements: the ZBTB32^+/+^ mice had an average body weight of 23,76 g compared to an average body weight of 26,05 g in ZBTB32^−/−^ mice (P = 0.0794). Food and water intake, O_2_ consumption, heat production and ambulatory activity were continuously recorded. Respiratory exchange ratio (RER) and energy expenditure were calculated. To exclude new cage environment bias, measurement of 72h of which the last 48h were used.

#### Real-time qPCR

Total liver RNA was isolated using the Aurum total RNA mini kit (Bio-Rad) according to the manufacturer's protocol. To generate cDNA (1000 ng of RNA was used), the Sensifast cDNA synthesis kit (GC Biotech BV) and dilutes 20 using ultrapure water for RT-qPCR. SensiFAST Sybr no-ROX mice (Bioline) were used to perform an RT-qPCR reaction in duplicate on a Roche LightCycler 480 (Applied Biosystems). RT-qPCR primers are listed in Table S1. *Hprt* and *Rpl* were used as housekeeping genes and relative expression of targeted genes was calculated via qBase + software (Biogazelle, Ghent, Belgium). Data was shown as means ± SEM.

#### Biochemical analysis

Plasma, liver, and adrenal gland free cholesterol levels were determined via the colorimetric Cholesterol Qquantitation Kit (Sigma), according to the manufacturer’s protocol while cholesterol ester was measured using the Cholesterol/Cholesteryl Ester Assay Kit (Abcam, ab65359). Blood glucose levels were measured in tail blood with the use of the OneTouch Verio glucometer (LifeScan). Free fatty acid (Abnova) and glycerol (Cayman Chemical) were measured in mouse plasma with the use of colorimetric assays according to manufacturer's instructions. GC (Corticosterone Enzyme Immunoassay kit, Harbor Assay) and ACTH (ACTH ELISA kit, Antibodies-online) levels were quantified via ELISA according to the manufacturer’s protocol in mouse plasma.

#### LipidTOX

Liver tissue (1cm³) was fixated in Antigen Fix buffer (DiaPath) for 2h at RT, followed by 10h–24h incubation in 34% sucrose at 4°C to maintain tissue morphology. Next, tissues were rinsed in PBS and embedded in Neg-50 Kryo-Medium. Liver section of 20 μm thick was rehydrated in PBS for 5 min. Then, blocking buffer (2% BSA, 1% fetal calf serum, and 1% goat serum in 0.5% saponin) was used to block the sections for 30 min at RT. After blocking, the antibody mix (LipidTOX Deep Red [1:400, Life Technologies Europe B.V.]; Acti-stain 488 Phalloidin [1:150, Cytoskeleton Inc.]) was added and incubated for 2h at RT after which the samples were washed with PBS for 5 min. The nuclei were then stained using Hoechst (1:1000, Sigma - Aldrich N.V.) for 5 min at RT. Slides were washed in PBS for 5 min, quickly rinsed in water to remove residual salt and mounted. Using the spinning disk confocal microscope (40x Plan-Apochromat objective lens [1.4 Oil DIC (UV) VIS-IR M27]) at a pixel size of 0.167 μm and at optimal Z-resolution (240 mm)), Z-stacks of 5–10 areas per cryosection were imaged. These Z-stacks were processed in Volocity (PerkinElmer) and the amount of lipid droplets and average size of lipid droplets (depicted as voxels) was determined.

#### Oil Red O staining

ZBTB32^+/+^ and ZBTB32^−/−^ mice were fed and starved 24h (n = 5/group). Adrenals were harvested from euthanized mice and embedded in O.C.T. medium (Sakura). Adrenal gland cryostat sections of 10 μm in thickness were rinsed with PBS after which the sections were fixated for 30 min at RT in 4% PFA. After washing with PBS for 1 min and absolute propylene glycol (Acros Organics AC158720010) for 5 min at RT, slides were stained with a pre-warmed 0.5% stock solution of Oil Red O (Sigma O-0625) at 60°C. Then, the slides were de-stained in 85% propylene glycol for 5 min on a shaker at RT and washed for 5 min with running tap water. After washing the slides, a counterstaining with Mayer’s Hematoxylin (Sigma) for 10 s at RT was performed. Again, the slides were washed with running tap water for 5 min and mounted with aquatex. Slides were scanned with the Zeiss slide scanner and pictures studied using Zeiss Zen software are displayed at 35% magnification.

#### Lipase activity assay

Activity of lipases in adipose tissue was analyzed via the Lipase Activity Assay Kit (Cayman Chemical). Epidydimal white adipose tissue (eWAT) was dissected, homogenized, and tested for lipolytic activity according to the manufacturer’s protocol.

#### MAPPIT(mammalian protein-protein interaction trap)

To identify novel GR interaction partners, the high-throughput technology MAPPIT, which had already been established in human embryonic kidney T (HEK293T) cells, was used. Independent MAPPIT screens were performed to evaluate the interactome of the GR in its inactive (absence of ligand) and active (presence of ligand) form. As bait in the MAPPIT screens, 25 ng of the plasmid pCLG-human GRa, encoding the full human GRα, was transfected in the HEK293T cells (using the calcium-phosphate method). The human ZBTB32, acquired from the human orfeome V5.1 collection, was also cloned in the pCLG vector and was used as prey and 50 ng was used to transfect the HEK293T cells. A STAT3-responsive rPAP-Luciferase reporter, of which 5 ng was transfected in HEK293T cells, was used to detect interaction. Cells were stimulated with vehicle or 1μM Dex for 24h and an empty vector was used as negative control.

#### Reporter gene assay

For the reporter assays, A549 cells were stably transfected with a GRE-Luc plasmid, as previously described in Dendoncker et al. (2017) (Dendoncker et al., 2017), using Lipofectamine and Lipofectamine Plus reagent according to the manufacturer’s instructions. Briefly, the luciferase reporter construct was driven by a synthetic GR-responsive promoter region containing two classic consensus GRE sequences (underlined) derived from the tyrosine aminotransferase (TAT) gene promotor AGATCTCTCTGCTGTACAGGATGTTCTAGCGGATCCTGCTGTACAGGATGTTCTAGCTACCTGCAG succeeded by a minimal IL6 promoter TATA box and followed by the luciferase gene of which the quantified luciferase expression is a direct measure of GR transcriptional activity. A549 cells stably transfected with GRE-Luc were transfected with a plasmid encoding flag-ZBTB32 at concentrations of 100 ng and 400 ng as indicated. Twenty-four hours after transfection, the medium was replaced by Optimem for cell starvation and 48h after transfection, the cells were stimulated with 1 μM Dex for 6h. Luciferase activity was measured using the Luciferase Assay System kit (Promega) on an Envision plate reader (PerkinElmer). The luciferase values are normalized to β-galactosidase activity. Graphs show the mean ± SEM of experiments performed in triplicate.

#### Co-immunoprecipitation and Western blotting HEK293T

GRα, inserted into the pMet7-CFP vector, and ZBTB32, cloned in the pMet-Flag vector, were used for co-immunoprecipitation (co-IP) experiments. HEK293T cells were seeded in 10 cm Petri dishes and transfected with the 4 μg of CFP-GRα and 3μg of flag-ZBTB32 plasmids via the calcium phosphate method (ref). Cells were stimulated with vehicle or 1μM Dex for 2h and harvested and homogenized in lysis buffer A (10 mM HEPES pH 7.5, 1.5 mM MgCl2, 10 mM KCl, 0.5 mM DTT, 0.1% NP40, supplemented with protease inhibitor cocktail (Roche)). The samples were subjected to two freeze-thaw cycles (−70°C) after which lysates were cleared by centrifugation at 13.000 rpm at 4°C and incubated with 20 μL of Anti-Flag M2 Affinity Gel beads (Sigma Aldrich) overnight at 4°C. The beads were washed 4x in buffer A supplemented with 150 mM NaCl and 0.5% Triton X-100. Next, the beads were re-suspended in Laemmli buffer and boiled for 1 min at 95°C. Immunoprecipitates were used for immunoblot using antibodies against GR and the FLAG tag.

Cells were treated as described in the section *co-immunoprecipitation*. Protein samples containing 30 μg of protein were separated by electrophoresis in a 10%-gradient SDS polyacrylamide gel and transferred to nitrocellulose membranes (pore size, 0.45 μm). After blocking the membranes with a ½ dilution of Starting Block/PBST 0.1% (Thermo Fisher Scientific), membranes were incubated overnight at 4°C with a primary antibodies against Flag (1:1.000, F7425, Sigma) or GR (1:1000, H300, Santa Cruz Biotechnology). Blots were washed with PBST 0.1% and then incubated for 1h at room temperature with the secondary anti-rabbit HRP antibody (1:10.000, 111-035-144, Jackson Immunoresearch laboratories). Immunoreactive bands were visualized, detected, and quantified using an Amersham Imager 600 (GE Healthcare Life Sciences).

#### Western blot adrenal glands

For the detection of ZBTB32 on protein level, total protein was isolated from snap frozen adrenal glands (2 adrenals were pooled from 1 mouse) with RIPA lysis buffer, supplemented with protease inhibitor cocktail (Roche). 50 μg of protein per sample was separated by electrophoresis using an 8% gradient SDS-polyacrylamide gel. Then, the protein was transferred to a nitrocellulose membrane (pore size 0.45μm) followed by blocking of the membranes (½ dilution of block/PBST 0.1% (Thermo Fischer Scientific)). Next, the membranes were incubated overnight at 4°C with primary antibody against ZBTB32 (1/1000, ZBTB32 Aviva Systems Biology, catalog numberOACA06573). Blots were washed with PBST 0.1% and then incubated for 1h at room temperature with Amersham ECL anti-rabbit HRP conjugated IgG antibody (1:2000, HAF008, R&D Systems). Visualization and quantification of the immunoreactive bands was performed using Amersham Imager 600 (GE Healthcare Life Sciences). The antibody recognized ZBTB32 (53 kDa) in ZBTB32+/+ but not in ZBTB32−/− mice (see Figure S11).

#### Co-immunoprecipitation adrenal glands

For the identification if ZBTB32 as a GR-binding protein, protein lysates were prepared from snap frozen adrenal glands (2 adrenals pooled from 1 mouse) by using RIPA lysis buffer supplemented with protease inhibitor cocktail (Roche). Immobilized (using 2 mg/mL BSA) Dynabeads (50μL bead slurry, cat nr: 11204D, ThermoFisher Scientific) were added to the lysate and rotated for 1h at 4°C. Using the Dyna-Mag 2 magnet (ThermoFisher Scientific) the supernatant (precleared lysate) was separated from the beads and the protein concentration was measured via a Bradford protein assay (Bio-Rad). Then, 75 μg precleared lysate was combined with 5μL anti-GR antibody (cat nr: sc-8992, Santa Cruz Biotechnology) and rotated for 1h at 4°C. Immobilized Dynabeads (50μL bead slurry) were added to the mixture and rotated for another 2h at 4°C. The bead-mixtures were washed three times with protease inhibitor supplemented RIPA buffer and were denatured for 5′ at 95°C using a thermomixer (Eppendorf). The samples were subjected to WB analysis and anti-GR (1:1000, G5, sc-393232; Santa Cruz) and anti-ZBTB32 was used to assay the interaction between immunoprecipitated GR and ZBTB32.

#### ChIP-seq

The genome-wide binding of GR in treated with PBS or Dex, was performed as described by us previously (Dendoncker et al., 2019). Briefly, BWTG3 cells were treated with 1 μM Dex (Sigma, D-2915) for 1h. After stimulation, cells were fixed with 1% PFA for 10 min at room temperature with shaking in order to crosslink protein and DNA. To stop the reaction, 125 mM glycine was added followed by washing of the cells using PBS. Then, the cells were lysed in lysis buffer (0.1% SDS, 1% Triton X-100, 0.15 M NaCl, 1 mM EDTA and 20 mM Tris pH8) supplemented with proteinase inhibitors (Complete EDTA free Protease Inhibitor Cocktail Tablets, Roche). Cell pellets were sonicated using a Biorupter instrument (Diagenode) for 30 min with 30 s on/30 s off intervals. 100 μL of cell lysates was diluted 1:3 in incubation buffer (0.15% SDS, 1% Triton X-100, 0.15M NaCl, 1mM EDTA and 20mM HEPES) for immunoprecipitation (IP) with rabbit anti-GR antibody (5μg per IP; Santa Cruz Biotechnology, sc-8992, Europe). The lysates were incubated with the antibody for 2h at 4°C before adding BSA blocked nProtein sepharose beads (GE Healthcare). The next day, immunoprecipitated material was collected by centrifugation and washed in spin columns with 0.35 μM pore size filters (MiBiTec), prior to elution with 200 μL elution buffer (1 M NaHCO_3_ and 10% SDS) supplemented with NaCl, proteinase K (1 mg/mL) and RNAse A (20 μg/mL) and incubated for 2h at 55°C and overnight at 65°C to de-crosslink the material. By using the PCR purification kit (Qiagen) according to the manufacturer's protocol, the DNA was purified in 50 μL Qiagen elution buffer. DNA libraries were sent to VIB nucleomics core for sequencing (Illumine HiSeq). Sequences were 52 cycle single read. The mm10 mouse genome was used to align the sequence reads (52 bases 54–69 million quality-filtered reads per sample) to the mouse genome using the bowtie without allowing a mismatch in the seed sequence. The number of unique alignments ranged from 39.4 million to 48.6 million. Only high-quality peaks (p < 0.0001 & FC > 4 over local background and input control) that were shared between all biological triplicates were retained.

#### ChIP-qPCR

ZBTB32^+/+^ and ZBTB32^−/−^ mice were injected intraperitoneally with Dex (200 μg/20g) or PBS (control), and 2h later adrenal glands from 3 mice were pooled which represent 1 biological replicate (∼20 mg tissue). Tissue was homogenized in 1 mL of PBS (supplemented with protease inhibitors, Complete EDTA free Protease Inhibitor Cocktail Tablets, Roche) and tissue homogenates were fixed with 1% of PFA to crosslink protein and DNA for 10 min. The reaction was stopped with 125 mM glycine, after which the homogenates were washed with PBS and lysed in lysis buffer (see ChIP-seq for composition), supplemented with protease inhibitors. Tissue pellets were sonicated using a Bioruptor instrument (Diagenode) for 30 min with 30s on/30s off intervals. Immunoprecipitation (IP) was performed on 100 μL lysates diluted 1:3 in incubation buffer (0.15% SDS, 1% Triton X-100, 0.15M NaCl, 1 mM EDTA and 20 mM HEPES) with rabbit anti-GR antibody (5 μg per IP, Santa Cruz Biotechnology, sc-8992), rabbit anti-ZBTB32 (5 μg per IP, ZBTB32 Aviva Systems Biology, catalog numberOACA06573) or normal rabbit IgG as a control (0.6 μg per IP, Peprotech, 500-P00). Antibody was incubated with the lysates for 2h at 4°C, after which BSA-blocked nProteinA (GR) or nProteinG (ZBTB32) Sepharose beads (GE Healthcare) were added. Immunoprecipitated material was collected the next day by centrifugation and washed in spin columns with 0.35 μM pore size filters (MiBiTec), prior to elution with 200 μL elution buffer (1 M NaHCO_3_ and 10% SDS). Next, NaCl, proteinase K (1 mg/mL) and RNAse A (20 μg/mL) was added and the reaction was incubated for 2h at 55°C and overnight at 65°C, to de-crosslink the material. The recovered DNA was purified by the PCR purification kit (Qiagen) according to the manufacturer’s protocol and eluted in 50 μL of Qiagen elution buffer. RT-qPCR was performed on 2 μL of chromatin per reaction with the sensiFAST Sybr no-ROX mix (Bioline) using the Roche LightCycler 480 system (Applied Biosystems). Primer sequences for target GR binding sequences are listed in Table S1. Results were normalized to input chromatin and shown as the ratio of GR (H300) recruitment vs. IgG control. Data are shown as mean ± SEM (n = 3 biological replicates/group, 3 PCR reactions from single experiment of 3 biological replicates (pooled adrenals from 3 mice)).

#### Liver RNA sequencing

ZBTB32^+/+^ and ZBTB32^−/−^ mice were injected with PBS or 200 μg Dex (n = 3/group) and 2h later, liver was removed and total RNA was isolated with Aurum total RNA mini kit (Biorad) according to manufacterer's instructions. RNA quality was checked with the Agilent RNA 6000 Pico Kit (Agilent Technologies). Using the Illumina TruSeqLT stranded RNA-seq library protocol (VIB Nucleomics Core), the RNA was used to make library preparations followed by single-end sequencing on the Illumina NextSeq 500. The mouse (mm10) reference genome/transcriptome was applied to map the obtained reads with HISAT v2.0.4 (Kim et al., 2015). Gene-level read counts were acquired with the featureCounts software (part of the subread package) (Liao et al., 2014). Multimapping reads were excluded from the assignment. The DESeq2 package was used to determine differential gene expression (Love et al., 2014), with the Fold Discovery Ratio (FDR) set at 5%. Transcription factor-binding sites on collections of transcripts were identified with the HOMER (v4.6) software (Heinz et al., 2010) and its accompanying collection of tools.

### Quantification and statistical analysis

All data are represented as mean ± SEM and analyzed using GraphPad Prism software. Statistical significance between groups was calculated using two-way Student’s t-tests, one-way ANOVA or two-way ANOVA with 95% confidence intervals. P < 0.05 was considered as significant (∗∗∗∗P < 0.0001; ∗∗∗P < 0.001; ∗∗P < 0.01; ∗P < 0.05). Body weight data over time were analyzed as repeated measurements using method of residual maximum likelihood (REML), as implemented in Genstat version 21. Because repeated measurements are taken from the same mouse, causing correlations among observations, a variance-covariance structure for the residual error term needs to be specified. The antedependence of order 2 (ANTE2) was finally selected as most appropriated variance-covariance structure based on the Akaike’s information criterion coefficient. The significance of the fixed terms in the model and significance of changes in difference between genotype and sex effects over time were assessed using an approximate F-test as implemented in Genstat version 21.

## Supplemental information

Document S1. Figures S1–S11 and Tables S1 and S3

Table S2. Identity of genes of Figure 6F and Gene Ontology pathways, related to Figure 6
