# Supplementary material for: ZBTB32 performs crosstalk with the glucocorticoid receptor and is crucial in glucocorticoid responses to starvation
Source: iScience. 2021 Jun 28;24(7):102790. doi: 10.1016/j.isci.2021.102790 (PMC8324811; doi:10.1016/j.isci.2021.102790)
Supplement: Document S1. Figures S1–S11 and Tables S1 and S3 [file mmc1.pdf]

iScience, Volume *24*

Supplemental information

ZBTB32 performs crosstalk with the glucocorticoid receptor and is crucial in glucocorticoid responses to starvation

Lise Van Wyngene, Tineke Vanderhaeghen, Ioanna Petta, Steven Timmermans, Katrien Corbeels, Bart Van der Schueren, Jolien Vandewalle, Kelly Van Looveren, Charlotte Wallaeys, Melanie Eggermont, Sylviane Dewaele, Leen Catrysse, Geert van Loo, Rudi Beyaert, Roman Vangoitsenhoven, Toshinori Nakayama, Jan Tavernier, Karolien De Bosscher, and Claude Libert

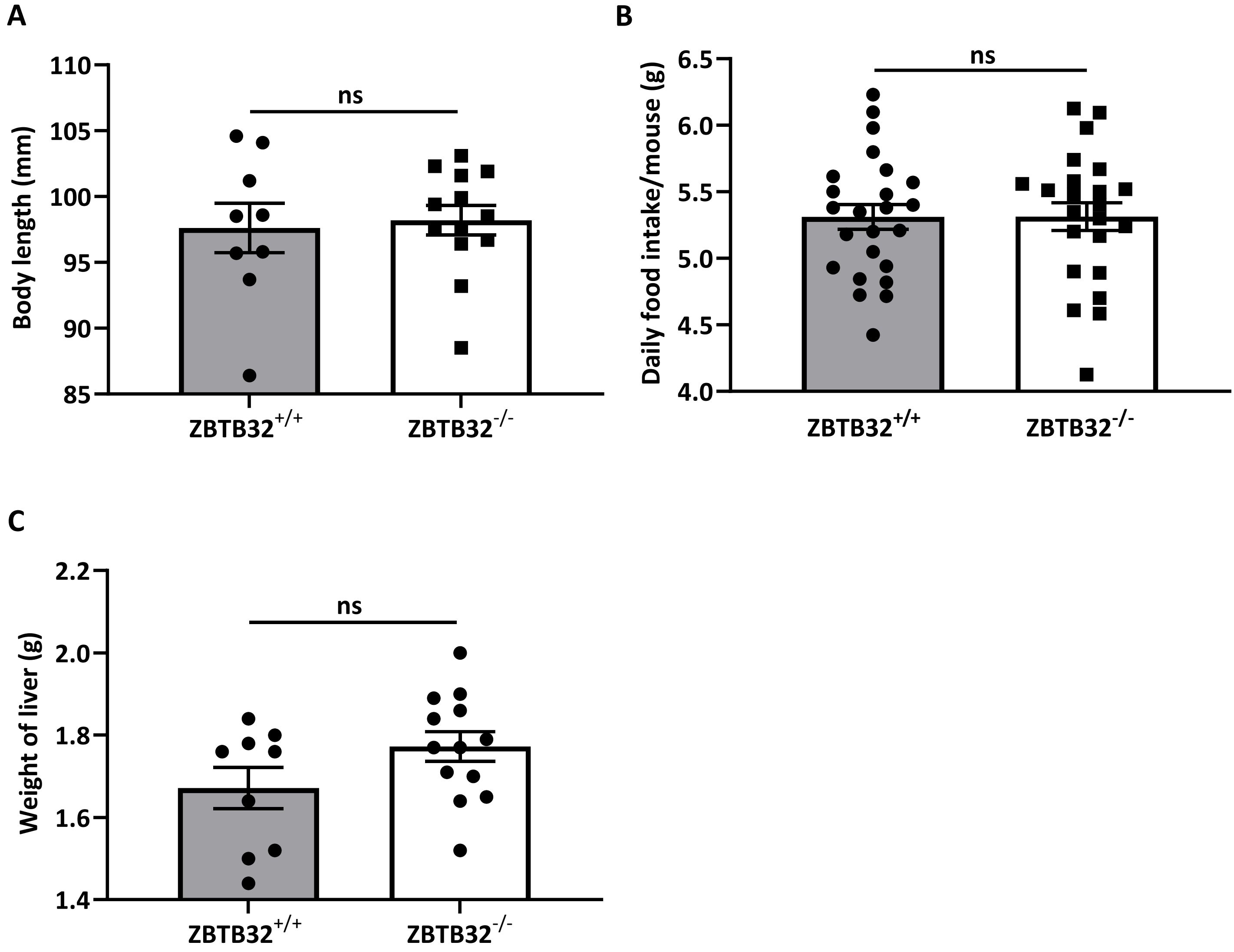

Figure S1. Increased weight of ZBTB32$^{-/-}$ mice is not caused by increased body length, food intake or organ mass (Related to Figure 1). Male ZBTB32$^{+/+}$ and ZBTB32$^{-/-}$ mice were aged to 25 weeks. **(A)** Average body length of mice at the age of 25 weeks, **(B)** average daily food intake of mice, followed between the age of 20 and 25 weeks, and **(C)** average weight of livers of mice at the age of 25 weeks. Values are shown as mean ± SEM, p-values were calculated via two-way Student's t-tests (n = 10-15 mice/group, pooled data of 2 independent experiments). ****P < 0.0001; ***P < 0.001; **P < 0.01; *P < 0.05.

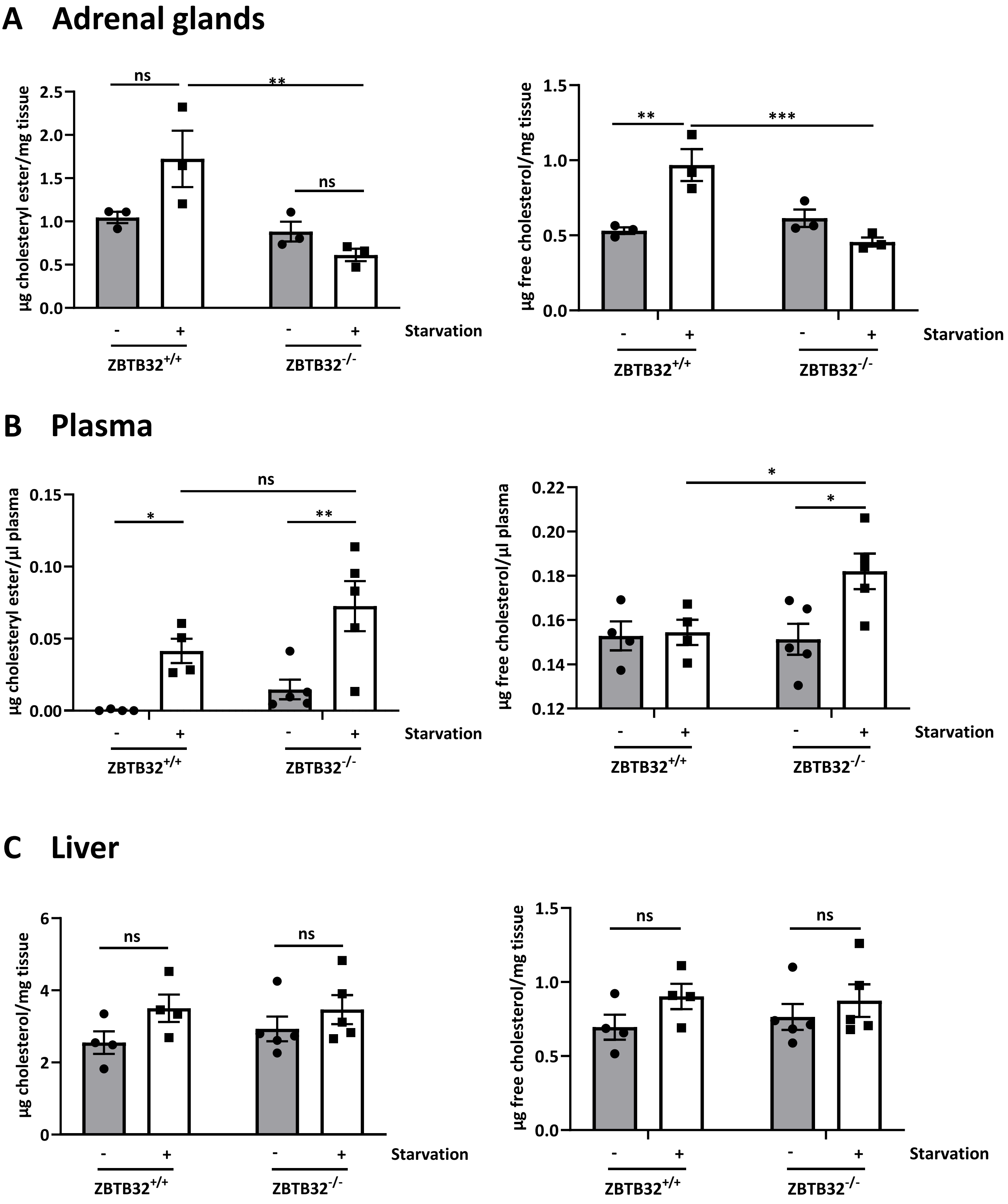

Figure S2. The levels of cholesterol esters and free cholesterol in fed and starved mice (Related to Figure 3). Male ZBTB32$^{+/+}$ and ZBTB32$^{-/-}$ mice of 12 week of age were fed *ad libitum* or starved for 24h, after which blood, liver and adrenal glands were isolated. Levels of cholesterol esters and free cholesterol in **(A)** adrenal homogenates, **(B)** plasma and **(C)** liver homogenates were quantified. Data are shown as mean ± SEM. P-values were determined via two-way ANOVA tests. ****P < 0.0001; ***P < 0.001; **P < 0.01; *P < 0.05.

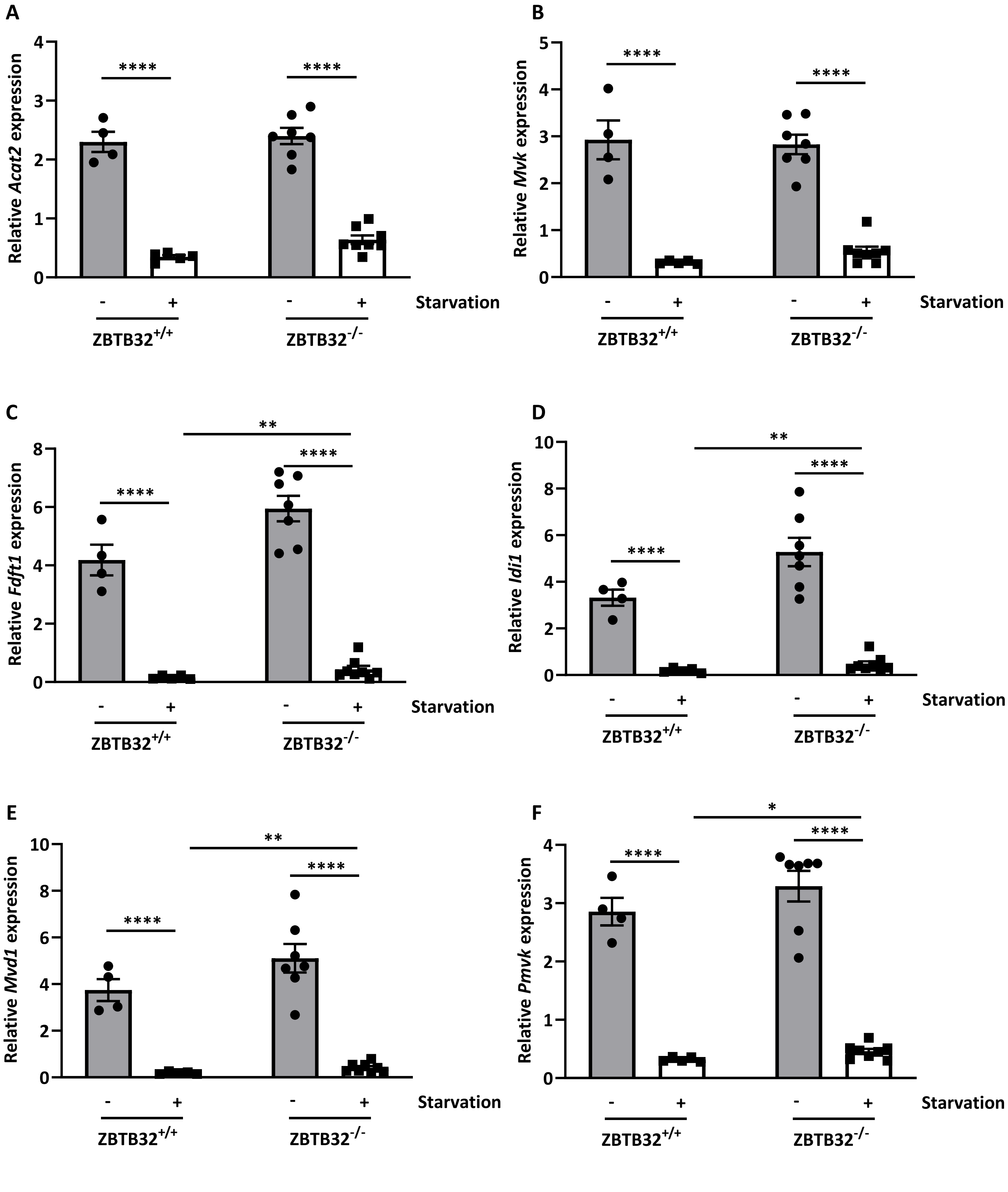

Figure S3. Cholesterol synthesis by the liver is not affected in ZBTB32$^{-/-}$ mice (Related to Figure 3). (A-F) ZBTB32$^{+/+}$ and ZBTB32$^{-/-}$ mice were fed *ad libitum* or starved for 24h (n=5-7 mice/group, data representative of 2 independent experiments). Liver was isolated and mRNA expression levels of cholesterol synthesis genes were determined via RT-qPCR. Data are shown as mean relative expression ± SEM. P-values were determined via two-way ANOVA tests. ****P < 0.0001; ***P < 0.001; **P < 0.01; *P < 0.05.

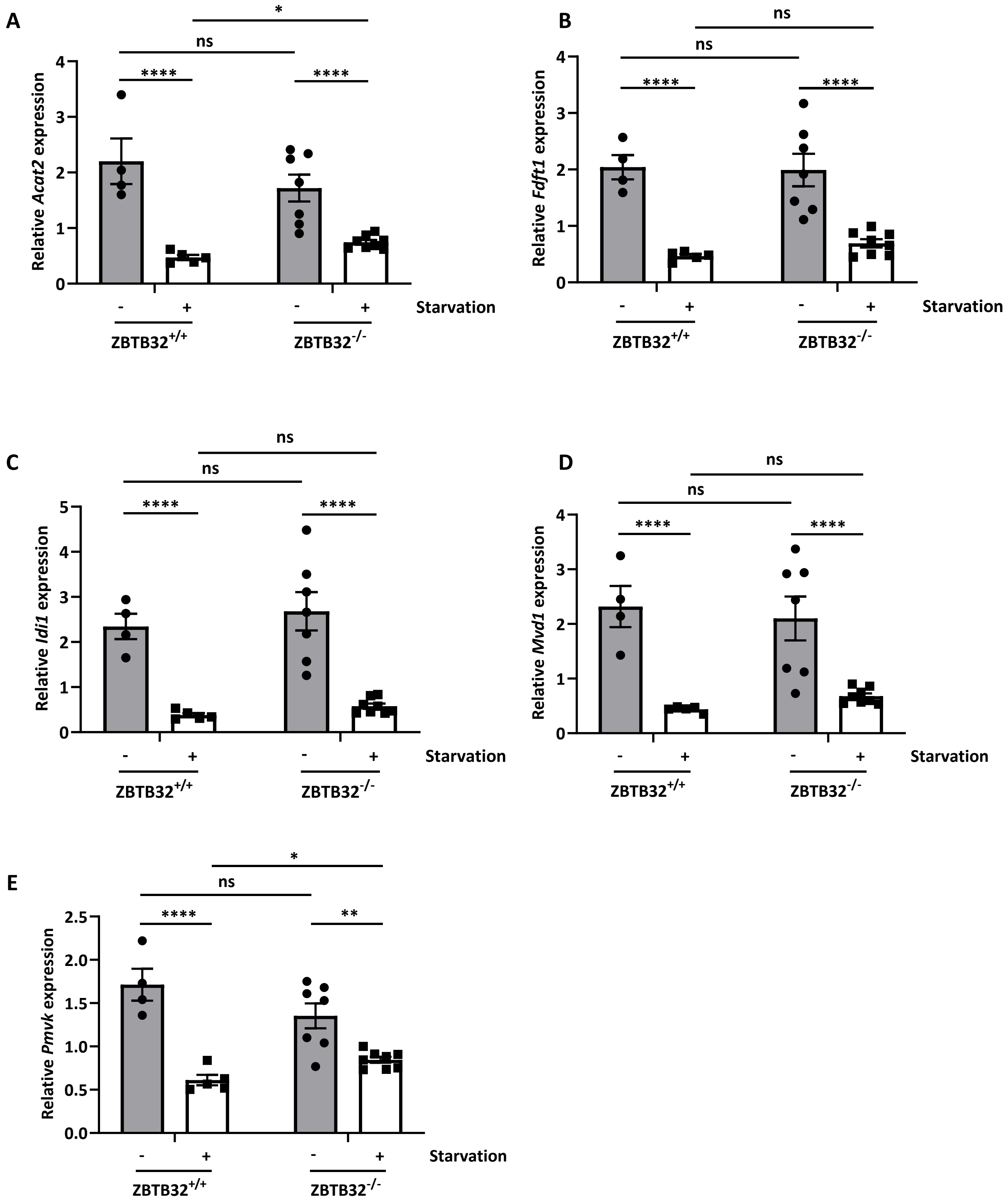

Figure S4. Cholesterol synthesis by the adrenal is not affected in ZBTB32$^{-/-}$ mice (Related to Figure 3). (A-E) ZBTB32$^{+/+}$ and ZBTB32$^{-/-}$ mice were fed *ad libitum* or starved for 24h (n=4-7 mice/group, data representative of 2 independent experiments). Adrenal glands were isolated and mRNA expression levels of cholesterol synthesis genes were determined via RT-qPCR. Data are shown as mean relative expression ± SEM. P-values were determined via two-way ANOVA tests. ****P < 0.0001; ***P < 0.001; **P < 0.01; *P < 0.05.

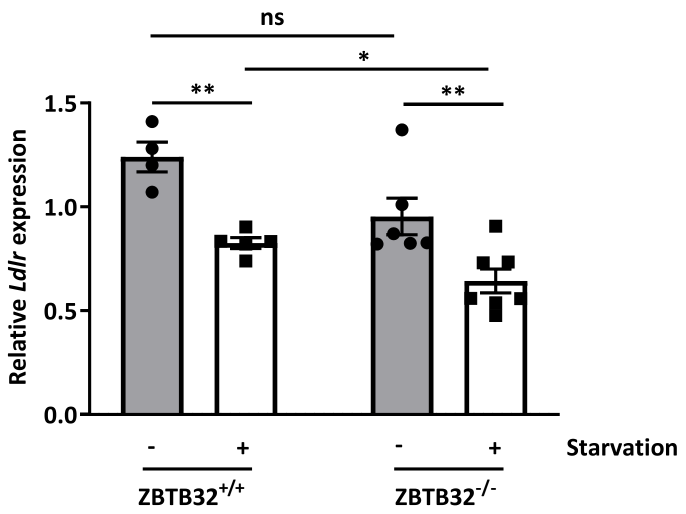

Figure S5. Ldlr gene expression in the adrenal of ZBTB32$^{+/+}$ and ZBTB32$^{-/-}$ mice (Related to Figure 4). ZBTB32$^{+/+}$ and ZBTB32$^{-/-}$ mice were fed *ad libitum* or starved for 24h (n=4-7 mice/group). Adrenals were isolated and mRNA expression levels of *Ldlr* were determined via RT-qPCR. Data are shown as mean relative expression ± SEM. P-values were determined via two-way ANOVA tests. **P < 0.01; *P < 0.05.

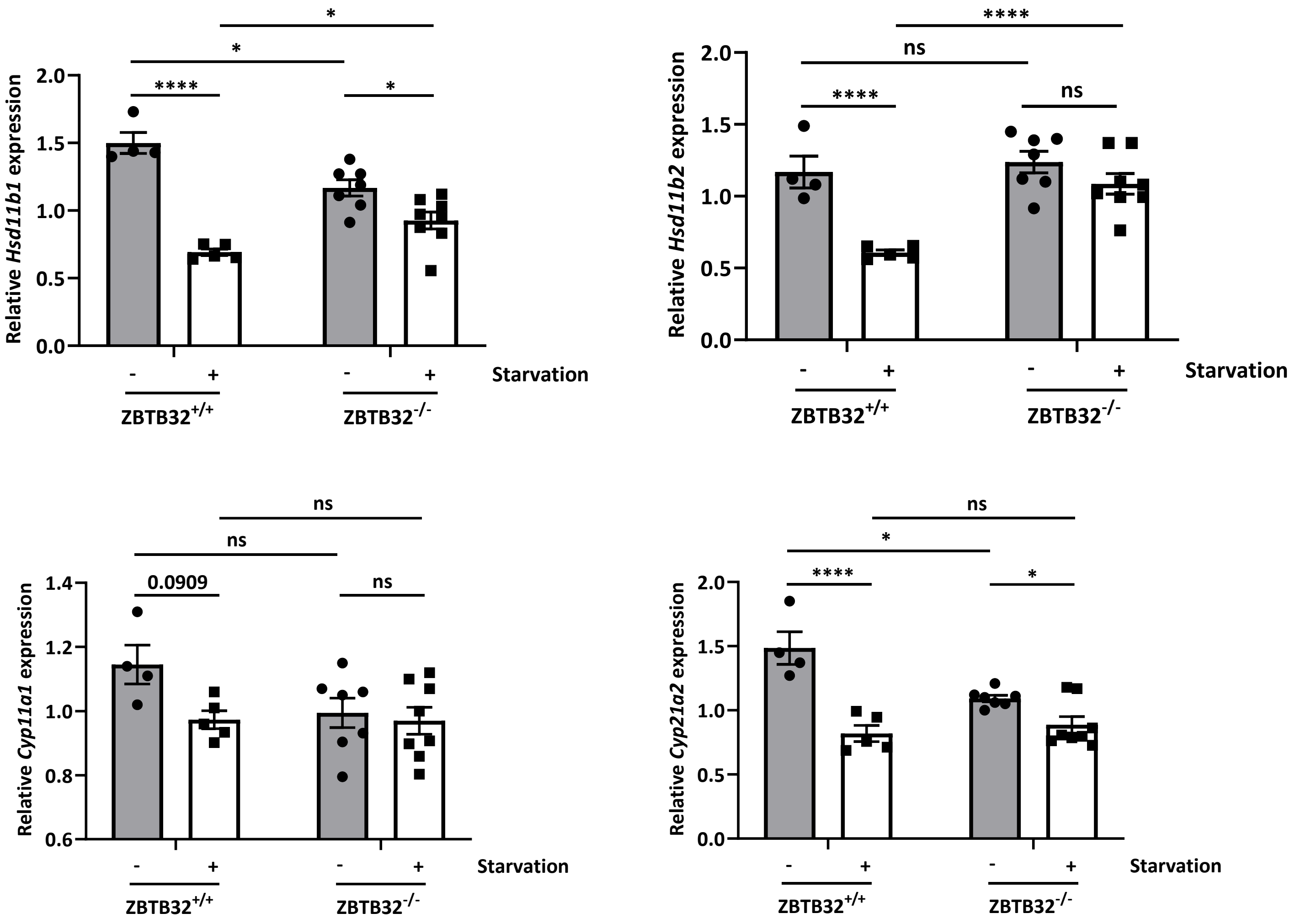

Figure S6. Genes involved in GC synthesis by the adrenals (Related to Figure 4). (A-E) ZBTB32$^{+/+}$ and ZBTB32$^{-/-}$ mice were fed *ad libitum* or starved for 24h (n=5-8 mice/group). Adrenals were isolated and mRNA expression levels of GC synthesis genes were determined via RT-qPCR. Data are shown as mean relative expression ± SEM. P-values were determined via two-way ANOVA tests. ****P < 0.0001; ***P < 0.001; **P < 0.01; *P < 0.05.

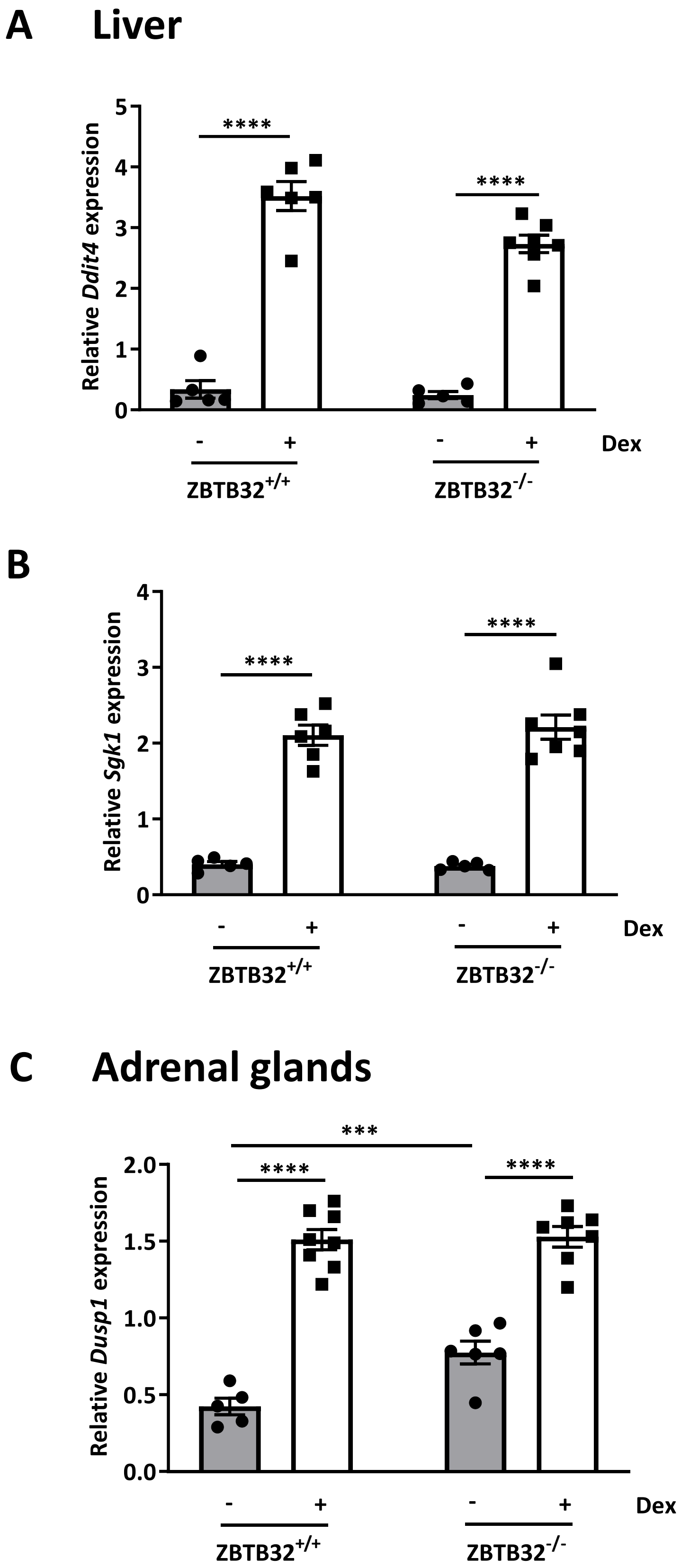

Figure S7. Expression of known GR-responsive genes in liver and adrenal glands is not affected by the absence of ZBTB32 (Related to Figure 4). (A-C) ZBTB32$^{+/+}$ and ZBTB32$^{-/-}$ mice were injected with Dex (200 µg/20 g) intraperitoneally, after 2h adrenal glands and liver were isolated. The expression of **(A)** hepatic *Ddit4* and **(B)** *Sgk1*, and **(C)** adrenal *Dusp1* was analyzed via RT-qPCR (n = 5-8 mice/group, data representative of 2 independent experiments). Values are shown as mean relative expression ± SEM. P-values were determined via two-way ANOVA tests. ****P < 0.0001; ***P < 0.001; **P < 0.01; *P < 0.05.

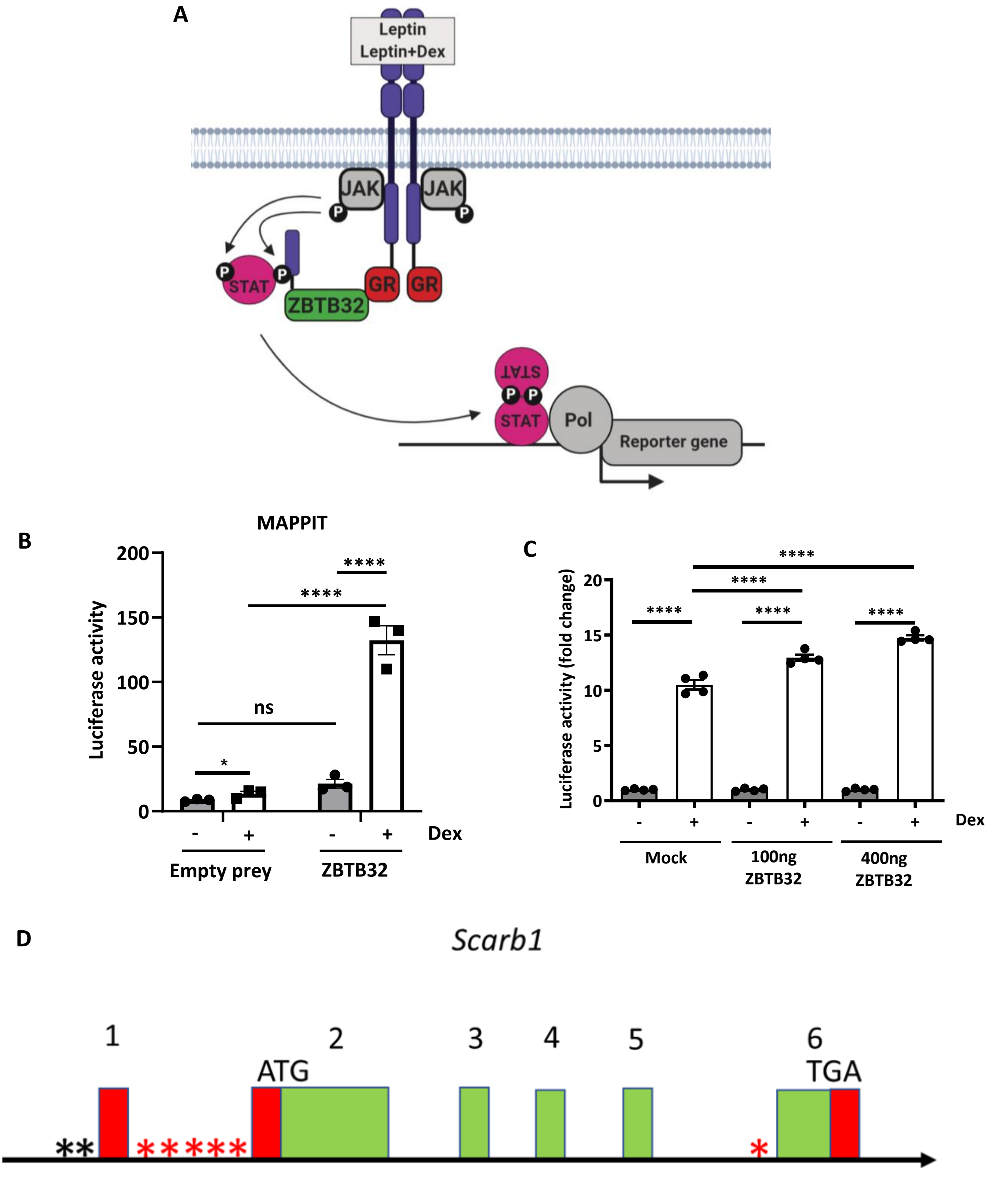

Figure S8. Identification of ZBTB32 as an interactor of GR via MAPPIT (Related to Figure 4). (A) Schematic representation of the Mammalian Protein-Protein Interaction Trap (MAPPIT) technology. Glucocorticoid receptor (GR) is fused to a mutated Leptin receptor that is unable to recruit STAT3. The ZBTB32-prey is bound to a gp130 fragment that contains functional STAT3 recruitment sites. Only upon interaction of GR with ZBTB32 and stimulation of with Leptin or Leptin + Dexamethasone (Dex), the Leptin receptor signaling cascade is reconstituted and STAT3 will be activated through phosphorylation. Next, STAT3 translocates to the nucleus and activates transcription of the luciferase reporter gene. **(B)** For the confirmation MAPPIT experiments, HEK293T cells were transfected with 25 ng GR-bait, 50 ng of ZBTB32 prey and 5 ng of rPAP1-Luc reporter and stimulated with vehicle (media) or with 100 ng/ml leptin or with 1 μM Dex and 100 ng/ml leptin for 24h (combined data of leptin and Dex+leptin treatment). The graphs represent the fold change induction to the vehicle-treated condition (no leptin) (triplicate wells from three independent experiment). **(C)** Luciferase expression induced by Dex treatment of GRE-Luc stably containing A549 cells, either mock transfected with empty vector, or transfected with 100 ng or 400 ng ZBTB32-expressing plasmid. P-values were determined via one-way ANOVA tests. **(D)** Schematic representation of potential GR-Binding sites (indicated as *) in the Scarb1 mouse gene. Binding sites in red were found by ChIP-SEQ and these in black by ConTraV3 analysis. Values are shown as mean ± SEM. P-values were determined via two-way ANOVA tests except if otherwise stated. ****P < 0.0001; ***P < 0.001; **P < 0.01; *P < 0.05.

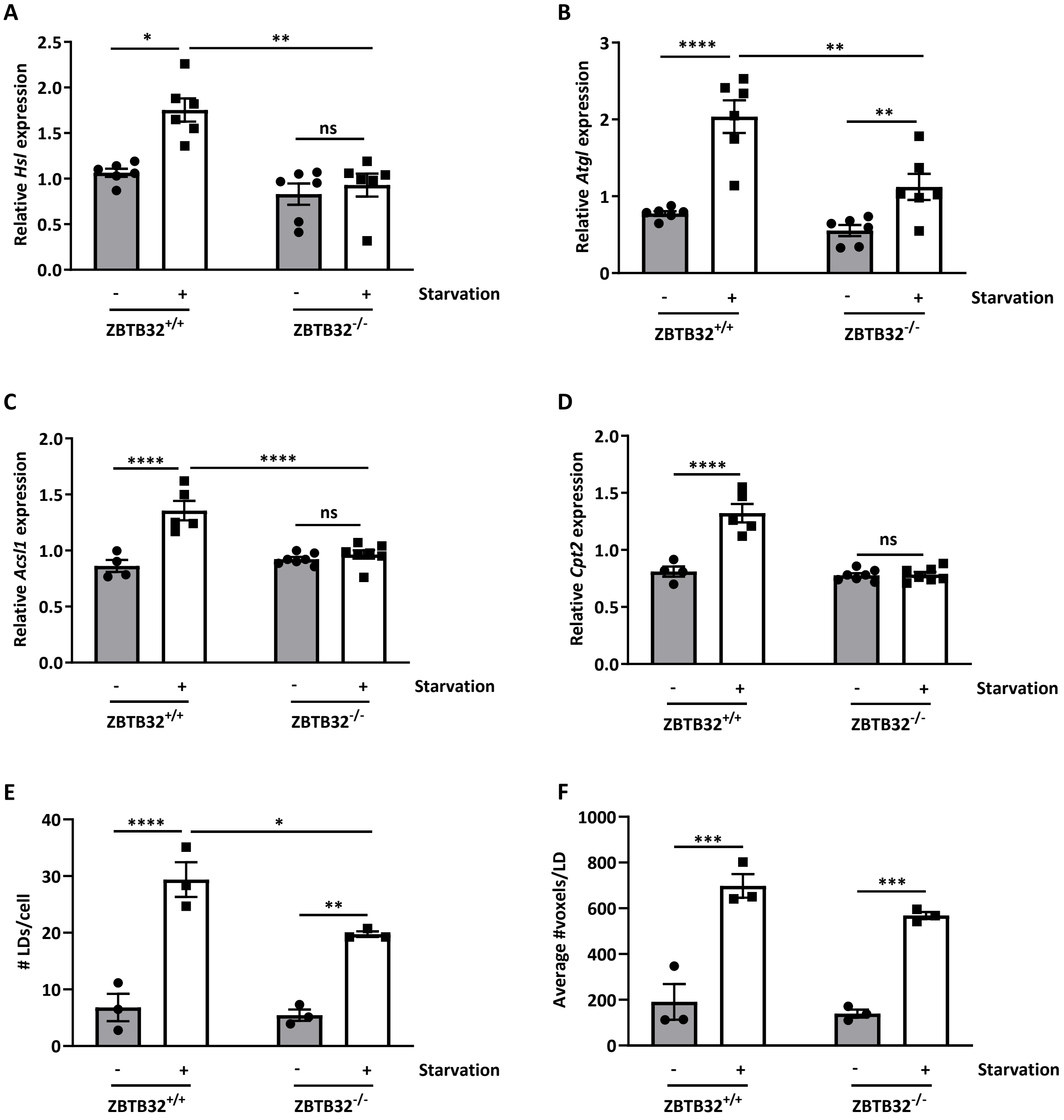

Figure S9. Defective corticosterone production leads to metabolic abnormalities during starvation in ZBTB32$^{-/-}$ mice (Related to Figure 5). ZBTB32$^{+/+}$ and ZBTB32$^{-/-}$ mice were fed ad libitum or starved for 24h (n = 5-7 mice/group, data representative of 2 independent experiments). **(A-B)** Epidydimal white adipose tissue (eWAT) was isolated and mRNA expression levels of **(A)** *Hsl* and **(B)** *Atgl* were determined via RT-qPCR. **(C-D)** Liver was isolated and mRNA expression levels of **(C)** *Acsl1* and **(D)** *Cpt2* were determined via qPCR. **(E-F)** Immunofluorescent images of livers visualizing lipid droplets (LDs). Cryosections were stained with Acti-stain (green), Hoechst (blue), and LipidTOX (red). Z-stacks were generated in 5-10 areas scattered across the entire tissue section. **(E)** The amount of lipid droplets (LDs)/cell and **(F)** average size of LDs (represented by voxel counts) were calculated for each Z-stack. Averages of the amount and size of LDs were converged for each mouse and biological replicates. Data are shown as mean ± SEM. P-values were calculated using two-way ANOVA. ****$P < 0.0001$; ***$P < 0.001$; **$P < 0.01$; *$P < 0.05$.

117

Total Target Sequences = 112, Total Background Sequences = 30256

Rank	Motif	Name	P-value	log P-pvalue	q-value (Benjamini)	# Target Sequences with Motif
1	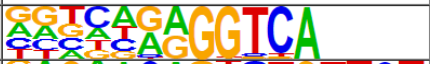	EAR2(NR)/K562-NR2F6-ChIP-Seq(Encode)/Homer	1e-4	-1.002e+01	0.0195	70.0
2	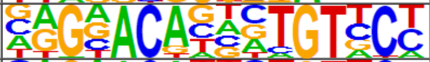	GRE(NR),IR3/A549-GR-ChIP-Seq(GSE32465)/Homer	1e-4	-9.421e+00	0.0195	15.0
3	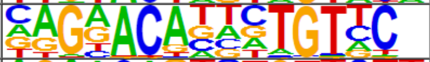	GRE(NR),IR3/RAW264.7-GRE-ChIP-Seq(Unpublished)/Homer	1e-3	-9.170e+00	0.0195	20.0
4	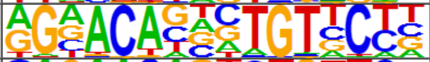	ARE(NR)/LNCAP-AR-ChIP-Seq(GSE27824)/Homer	1e-3	-8.765e+00	0.0195	21.0
5	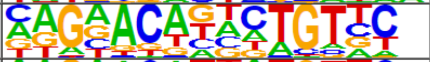	PR(NR)/T47D-PR-ChIP-Seq(GSE31130)/Homer	1e-3	-7.718e+00	0.0391	83.0
6	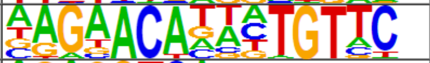	PGR(NR)/EndoStromal-PGR-ChIP-Seq(GSE69539)/Homer	1e-2	-6.627e+00	0.0971	17.0
7	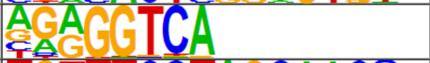	COUP-TFII(NR)/Artia-Nr2f2-ChIP-Seq(GSE46497)/Homer	1e-2	-5.086e+00	0.3885	73.0
8	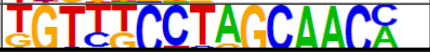	Rfx6(HTH)/Min6b1-Rfx6.HA-ChIP-Seq(GSE62844)/Homer	1e-2	-4.994e+00	0.3885	59.0

236

Total Target Sequences = 226, Total Background Sequences = 28439

Rank	Motif	Name	P-value	log P-pvalue	q-value (Benjamini)	# Target Sequences with Motif
1	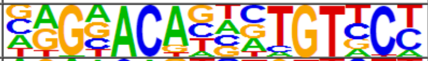	GRE(NR),IR3/A549-GR-ChIP-Seq(GSE32465)/Homer	1e-4	-1.127e+01	0.0056	25.0
2	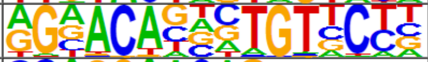	ARE(NR)/LNCAP-AR-ChIP-Seq(GSE27824)/Homer	1e-4	-1.029e+01	0.0075	36.0
3	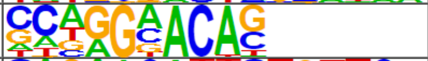	AR-halfsite(NR)/LNCaP-AR-ChIP-Seq(GSE27824)/Homer	1e-3	-8.725e+00	0.0238	208.0
4	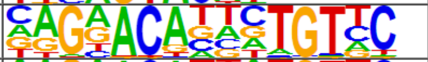	GRE(NR),IR3/RAW264.7-GRE-ChIP-Seq(Unpublished)/Homer	1e-3	-8.674e+00	0.0238	32.0
5	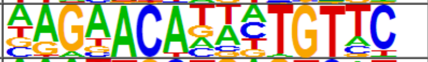	PGR(NR)/EndoStromal-PGR-ChIP-Seq(GSE69539)/Homer	1e-3	-7.608e+00	0.0437	30.0
6	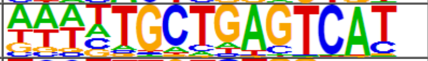	NFE2L2(bZIP)/HepG2-NFE2L2-ChIP-Seq(Encode)/Homer	1e-2	-5.202e+00	0.4038	8.0
7	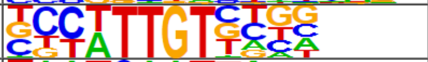	Sox21(HMG)/ESC-SOX21-ChIP-Seq(GSE110505)/Homer	1e-2	-5.059e+00	0.4038	136.0
8	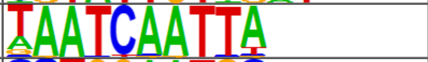	Pax7(Paired,Homeobox)/Myoblast-Pax7-ChIP-Seq(GSE25064)/Homer	1e-2	-4.941e+00	0.4038	12.0
9	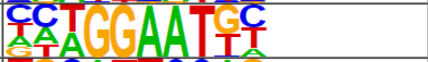	TEAD(TEA)/Fibroblast-PU.1-ChIP-Seq(Unpublished)/Homer	1e-2	-4.919e+00	0.4038	63.0
10	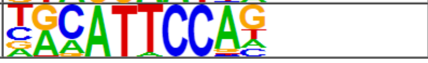	TEAD3(TEA)/HepG2-TEAD3-ChIP-Seq(Encode)/Homer	1e-2	-4.625e+00	0.4315	98.0

74

Known Motif Enrichment Results (txt file)

Total Target Sequences = 68, Total Background Sequences = 29177

Rank	Motif	Name	P-value	log P-pvalue	q-value (Benjamini)	# Target Sequences with Motif
1	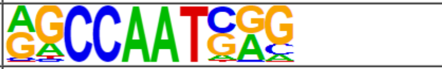	NFY(CCAAT)/Promoter/Homer	1e-2	-5.291e+00	1.0000	36.0
2	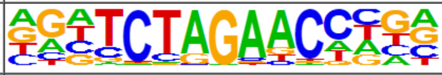	ZBTB12(Zf)/HEK293-ZBTB12.GFP-ChIP-Seq(GSE58341)/Homer	1e-2	-5.055e+00	1.0000	20.0
3	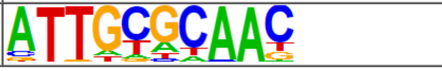	CEBP(bZIP)/ThioMac-CEBPb-ChIP-Seq(GSE21512)/Homer	1e-2	-4.943e+00	1.0000	24.0

Figure S10. Homer motif analysis of Dex-induced genes in ZBTB32$^{+/+}$ and ZBTB32$^{-/-}$ mice (Related to Figure 6). The enriched, known motifs, by Homer, are referring to the Venn diagram of Figure 6E. The group of 117 genes are genes induced by Dex in ZBTB32$^{+/+}$ mice only, the 236 genes are induced by Dex in ZBTB32$^{+/+}$ and ZBTB32$^{-/-}$ genes, and the group of 74 are Dex-induced only in ZBTB32$^{-/-}$ mice. Obviously, the groups of 117 and 236 genes are characterized by classical GRE element, but not the group of 74 genes.

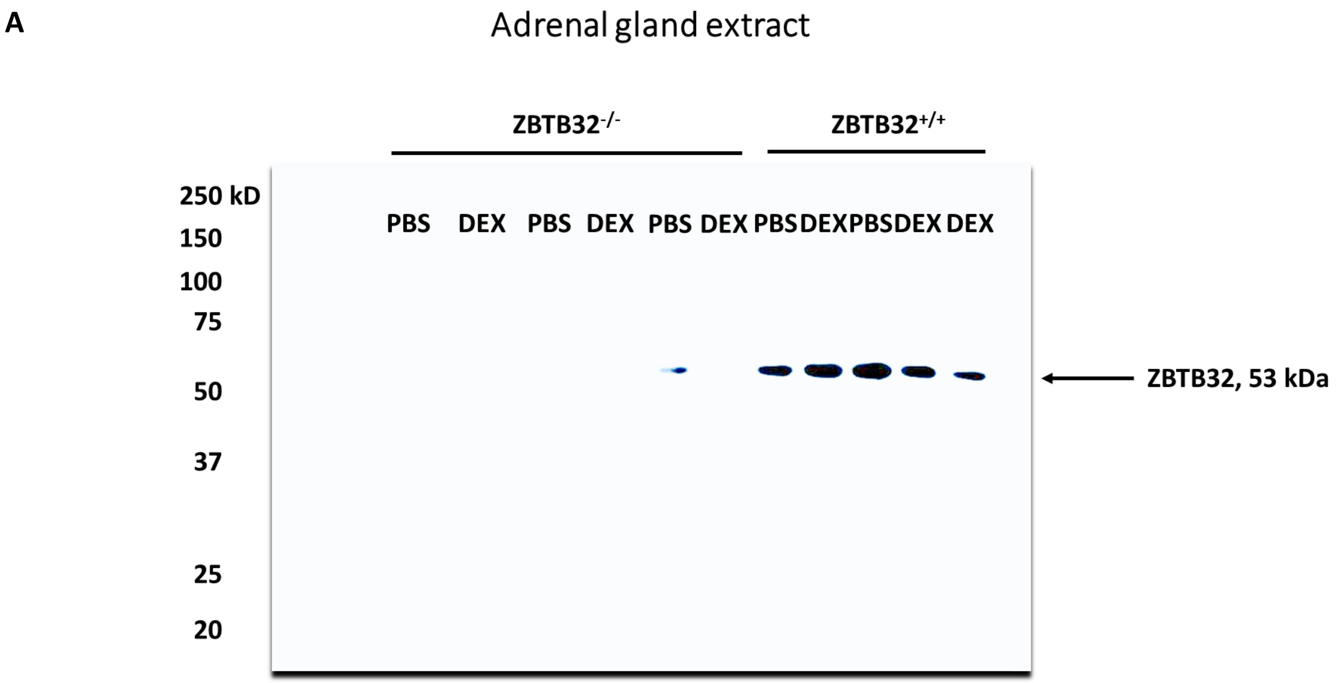

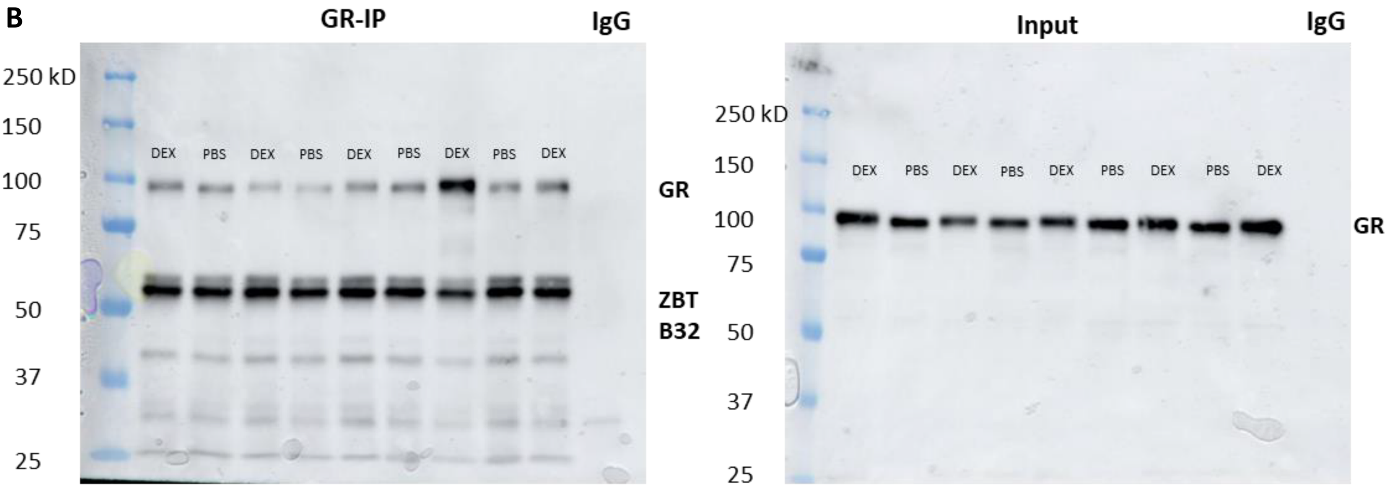

Figure S11. Western Blot analysis and co-IP on the adrenal glands of ZBTB32$^{+/+}$ and ZBTB32$^{-/-}$ after Dex stimulation (Related to Figure 4). (A) Western blot for ZBTB32 in adrenals of ZBTB32$^{+/+}$ and ZBTB32$^{-/-}$ mice. Both adrenal glands of each mouse were collected and pooled and minced and protein prepared, and 50 µg loaded on a polyacrylamide gel and blotted. The blots were probed using a rabbit polyclonal antibody, specifically validated to recognize ZBTB32 in mouse adrenals. The antibody recognized ZBTB32 (53 kDa) in ZBTB32$^{+/+}$ but not in ZBTB32$^{-/-}$ mice. **(B)** Western blots for GR and ZBTB32 (left) and GR (right) after GR-IP on adrenal glands of mice injected with PBS or Dex (200 ug) 2h earlier. A pool of all samples was treated with IgG control, to test GR specificity of the IP. Left, the results after GR-IP, right the input samples prior to IP.

Table S1: Overview of the sequences of the (ChIP) qPCR (Related to STAR Methods – Method details).

Gene Name	Forward primer (5' – 3')	Reverse primer (3' – 5')
Hprt	AGTGTTGGATACAGGCCAGAC	CGTGATTCAAATCCCTGAAGT
Rpl	CCTGCTGCTCTCAAGGTT	TGGTTGTCACTGCCTCGTACTT
Actin	GCTTCTAGGCGGACTGTAACTGA	GCCATGCCAATGTTGTCTCTTAT
PPARa	AGAGCCCCATCTGTCCTCTC	ACTGGTAGTCTGCAAAACCAAA
Acsl1	TGCCAGAGCTGATTGACATTC	GGCATACCAGAAGGTGGTGAG
Cpt1a	CTCCGCCTGAGCCATGAAG	CACCAGTGATGATGCCATTCT
Cpt2	CAGCACAGCATCGTACCCA	TCCCAATGCCGTTCTCAAAT
Acat2	CCCGTGGTCATCGTCTCAG	GGACAGGGCACCATTGAAGG
Mvk	GGTGTGGTCGGAACTTCCC	CCTTGAGCGGGTTGGAGAC
Fdft1	ATGGAGTTCGTCAAGTGTCTAGG	CGTGCCGTATGTCCCCATC
Idi1	AGCTTCTAGCGGAGATGTGTA	CAGCAACTATTGGTGAAACAACC
Mvd1	ATGGCCTCAGAAAAGCCTCAG	TGGTCGTTTTTAGCTGGTCCT
Pmvk	AAAATCCGGGAAGGACTTCGT	AGAGCACAGATGTTACCTCCA
Ldlr	TGACTCAGACGAACAAGGCTG	ATCTAGGCAATCTCGGTCTCC
Scarb1	TTTGGAGTGGTAGTAAAAAGGGC	TGACATCAGGGACTCAGAGTAG
Hsd11b1	CAGAAATGCTCCAGGGAAAGAA	GCAGTCAATACCACATGGGC
Hsd11b2	GGTTGTGACACTGGTTTTGGC	AGAACACGGCTGATGTCCTCT
Cyp11a1	AGGTCCTTCAATGAGATCCCTT	TCCCTGTAAATGGGGCCATAC
Cyp21a2	ACCTGTCCTTGGGAGACTAC	TTACCTCACAGAACTCCTGGGT
Ddit4	CAAGGCAAGAGCTGCCATAG	CCGGTACTTAGCGTCAGGG
Sgk1	GAGATCGTGTTAGCTCCAAAGC	CTGTGATCAGGCATAGCACACT
Dusp1	GTTGTTGGATTGTCGCTCCTT	TTGGGCACGATATGCTCCAG
Hsl	CCAGCCTGAGGGCTTACTG	CTCCATTGACTGTGACATCTCG
Atgl	CAACGCCACTCACATCTACGG	GGACACCTCAATAATGTTGGCAC

ChIP-qPCR primers

Scarb1_5' UTR1	TGGCAACCGGATCTGTTCTC	GCCTGACTCCTCCTCTCTCA
Scarb1_5' UTR2	GCGACCTCTTCTTTCTTCCG	TCCCGGGAGACATGGAATTC
Scarb_I1-3	CAGACGGCAAACATGTGGGTA	GCTGAGCTTCTGCTAGTGTC

Table S3: GR Interaction partners discovered by MAPPIT technology (Related to Figure 4). In this list, we show the novel GR-interaction partners discovered by us. We have picked up also known GR-interaction partners, that had been discovered by others, using other techniques, namely, HSP90AA1, HSP90AB15, HSP67, PPP5C8 , PTGES39, CREB111, HDAC812, STAT3, NR0B1, NR0B2, ZBTB9, SORBS3 and NCOA4.

Novel interaction partners of the activated GR		
**Abbreviation**	**Recommended name**	**Molecular function**
**FASTK**	Fas-activated serine/threonine kinase	Kinase activity, ATP-binding
**LPXN**	Leupaxin	Regulation of transcription
**ZBTB32**	Zinc finger and BTB domain-containing protein 32	Regulation of transcription
**LZTS2**	Leucine zipper putative tumor suppressor 2	Regulation of cell cycle
**RUSC1**	RUN and SH3 domain-containing protein 1	Protein polyubiquitination
**STOX1**	Storkhead-box protein 1	Transcription regulation, regulation of cell cycle
**CPNE5**	Copine-5	Membrane trafficking
**AHCYL1**	Adenosylhomocysteinase 2	L-homocysteine biosynthesis
**DAPK1**	Death-associated protein kinase 1	Serine/threonine-protein kinase
**C1orf94**	Uncharacterized protein C1orf94	-
**C9orf156**	Uncharacterized protein C9orf156	-
**RAI1**	Retinoic acid-induced protein 1	Regulation of transcription
**STRN**	Striatin	Scaffold protein
**STRN3**	Striatin-3	Scaffold protein
**GGA2**	ADP-ribosylation factor-binding protein GGA2	Protein transport
**NUP54**	Nucleoporin p54	Protein trafficking across nuclear membrane
**GTPBP3**	GTP-binding protein 3	GTPase activity
**PDLIM7**	PDZ and LIM domain protein 7	Cytoskeleton organization
**EIF4ENIF1**	Eukaryotic translation initiation factor 4E transporter	Regulation of translation, protein transporter
